# Effects of isometric training based on the entire population on blood pressure regulation: systematic review and meta-analysis of randomized controlled trials

**DOI:** 10.3389/fpubh.2026.1774541

**Published:** 2026-03-02

**Authors:** Yu Yan, Chu Sun, Lidan Pan, Hui Ma, Huisong Xie

**Affiliations:** 1 College of Education, Beijing Sport University, Beijing, China; 2 The School of International Education and Exchange, Beijing Sport University, Beijing, China; 3 Sports Coaching College, Beijing Sport University, Beijing, China

**Keywords:** cardiovascular, diastolic blood pressure, isometric exercise training, meta-analysis, systolic blood pressure

## Abstract

**Background:**

Although numerous studies have investigated the influence of isometric exercise on the management of resting blood pressure, consistent conclusions have not been reached. This study aims to assess the effect of isometric exercise on resting blood pressure regulation and to identify the key parameters of an effective training protocol through subgroup analysis, thereby providing a scientific foundation for individualized exercise prescriptions.

**Methods:**

Following the PRISMA guidelines, a systematic search was conducted across the PubMed, Web of Science, EBSCO, Cochrane, and Scopus databases. The search cutoff date was set for September 7, 2025. Emphasis was placed on randomized controlled trials published in the past decade.

**Results:**

A total of 40 randomized controlled trials were included. Meta-analysis results demonstrated that isometric training significantly reduced SBP (WMD, −6.62; 95% CI, −8.13 to −5.10, *p* < 0.0001, *I*^2^ = 75%) and DBP (WMD, −2.63; 95% CI, −3.50 to −1.76, *p* < 0.0001, *I*^2^ = 50%). Regression analysis revealed no significant influencing factors. Subgroup analyses suggested that, within the analyzed studies, larger reductions in blood pressure were observed in trials implementing wall squat exercises three times per week for a duration exceeding 8 weeks, particularly among males and hypertensive populations. The intensity associated with the largest effect size differed between systolic (85% HR peak) and diastolic (95% HR peak) blood pressure.

**Conclusion:**

Isometric exercise, particularly wall squats performed three times weekly for over 8 weeks, is associated with significant reductions in resting blood pressure. The greatest benefits were observed in males and hypertensive individuals. While higher intensities (e.g., 85–95% HR peak) are effective, the preferable intensity may differ between systolic and diastolic blood pressure and should be individualized. This training represents a valuable adjunctive therapeutic strategy.

**Systematic review registration:**

https://www.crd.york.ac.uk/PROSPERO/view/CRD420251171800, Identifier CRD 420251171800.

## 1 Introduction

Hypertension represents one of the most critical challenges in global public health (1). According to the Global Burden of Disease Study, hypertension-related conditions are responsible for more than 10% of the worldwide disease burden (2). Tight control of resting blood pressure is widely recognized as a fundamental strategy for preventing target organ damage and reducing the incidence of cardiovascular events. Conventional management strategies include both pharmacological and non-pharmacological interventions (3). Pharmacological treatments typically involve the use of antihypertensive agents—such as calcium channel blockers or angiotensin-converting enzyme inhibitors—tailored to individual patient profiles. Nevertheless, these medications may lead to adverse effects like peripheral edema and dry cough, require long-term adherence, and pose a risk of rebound hypertension upon discontinuation (4). Non-pharmacological approaches focus on lifestyle modifications, such as adopting a diet low in salt and fat but rich in potassium and fiber, maintaining a healthy body weight, quitting smoking, limiting alcohol consumption, ensuring regular sleep, and cultivating a positive mental state (5). However, sustained compliance with these measures remains difficult for many patients. In light of these limitations, there is an urgent need to explore more effective and sustainable treatment alternatives (6). Exercise therapy has emerged as a cornerstone of modern hypertension management due to its favorable cost-effectiveness and generally high adherence. Aerobic exercises, including running and swimming, improve oxygen uptake and enhance systemic circulation, contributing to blood pressure reduction (7). More recently, isometric exercise—a form of static physical activity involving sustained muscle contractions (8). In contrast to aerobic exercise, isometric training requires minimal movement and can be performed at moderate intensity, yet it exhibits distinct antihypertensive properties (9). Thus, it offers a promising therapeutic avenue for hypertension and may represent an innovative approach to blood pressure control.

Early studies have confirmed that regular isometric exercise can significantly reduce resting blood pressure. However, significant heterogeneity exists among the findings of available research. Carlson et al. (10) an eight-week study on isometric grip strength training, observing significant improvements in participants’ mean blood pressure, strongly supporting the antihypertensive efficacy of isometric exercise. Similarly, Cohen et al. (11) implemented a 12-week wall-squat training protocol in hypertensive patients and reported statistically significant decreases in both systolic and diastolic blood pressures. In contrast, Danielsen et al. (12) found in their 20-week isometric handgrip intervention that while participants’ physical activity capacity improved, no significant effect on resting blood pressure control was demonstrated. These inconsistent findings across studies highlight substantial heterogeneity in research outcomes, which primarily stems from variations in training protocols—including differences in exercise modality, intensity, weekly frequency, and total intervention duration.

Although several systematic reviews have attempted to synthesize the evidence regarding the effects of isometric exercise on blood pressure, significant limitations remain. Inder et al. (13), for instance, investigated the role of isometric exercise in blood pressure management but included only healthy adults, excluding hypertensive and sedentary populations, thereby limiting the generalizability of their findings to other demographic groups. Similarly, Carlson et al. (14) conducted a meta-analysis that incorporated studies only up to 2013 and involved a relatively small number of participants. With numerous recent studies emerging, their conclusions may now differ from current evidence. Furthermore, López et al. (15) investigated the impact of isometric resistance training on resting blood pressure in an adult cohort; however, their assessment remained limited to a fundamental evaluation of blood pressure variations, without exploring dose–response relationships such as the influence of exercise type, session duration, or weekly frequency. In another study, Baffour et al. (16) focused specifically on individuals with prehypertension and hypertension and adopted an isometric handgrip training protocol. While valuable for this specific population, the results may not be generalizable to other forms of isometric exercise or broader populations. In summary, most meta-analyses have not comprehensively addressed population diversity, which weakens the identification of isometric exercise’s specific effects. More importantly, few studies have thoroughly investigated the dose–response relationships for key training parameters—such as the combination of intensity, duration, and frequency—to determine which combinations are associated with maximal blood pressure reduction. This knowledge gap hinders the precise formulation of exercise prescriptions in clinical practice and impedes the broader application of isometric exercise in public health initiatives.

Therefore, this study aims to systematically screen randomized controlled trials worldwide to investigate the effect of isometric exercise training on resting blood pressure control. Specifically, the Population comprises adults; the Intervention is isometric exercise training; the Comparison is non- intervention or usual care; the Outcomes are changes in resting systolic and diastolic blood pressure; and the Study design is randomized controlled trials. The primary objectives are: first, to quantify the overall effect size of isometric exercise training on resting blood pressure, thereby clarifying its clinical efficacy as a non-pharmacological intervention; second, to explore and describe key training parameters (e.g., modality, intensity, frequency, duration) associated with greater blood pressure reductions through subgroup and meta-regression analyses, thereby informing the future development of individualized exercise prescriptions.

## 2 Materials and methods

### 2.1 Design

This study was conducted in accordance with the (PRISMA 2020) statement (17), and registered it in PROSPERO with the number of CRD 420251171800.

### 2.2 Search strategy

In accordance with the PRISMA guidelines, a comprehensive search was performed across PubMed, Web of Science, Embase, Cochrane, and Scopus to identify all relevant studies up to 7 September 2025, with an emphasis on randomized controlled trials published in the preceding decade. The search strategy employed a combination of MeSH terms and keywords, including “isometric exercise,” “resting blood pressure,” “hypertension,” “exercise training protocol,” and “blood pressure control.” In addition to electronic database searches, we manually screened the reference lists of included studies, with particular attention to highly cited reviews and meta-analyses published in the last 5 years. Unpublished clinical trials were tracked via the Cochrane Central Register of Controlled Trials. Two researchers, Y.Y. and C.S., independently carried out the search and study selection procedures. They utilized a standardized data extraction form to document the characteristics of each study. In cases where there were any discrepancies, they engaged in discussions with a third, more senior researcher, H.X., and continued until a unanimous agreement was achieved.

### 2.3 Eligibility criteria

Inclusion criteria comprise: (1) evidence from randomized controlled trials; (2) an intervention group performing isometric exercise training compared with a control group receiving non- intervention or usual care; (3) if participants were on antihypertensive medication, medication use was required to be stable throughout the trial and evenly distributed between groups; (4) adult (age ≥ 18 years) without restrictions on gender, health, or disease status, including those with prehypertension or hypertension without significant comorbidities, and no engagement in large-scale physical activity within 6 months prior to the trial; and (5) availability of resting blood pressure as an outcome measure. (6) Only studies implementing long-term isometric exercise training interventions were included. Studies assessing acute cardiovascular responses (i.e., single-session or immediate post-exercise effects) were excluded. The minimum intervention duration was defined as ≥1 week, with at least 3 total sessions conducted.

Exclusion criteria included: The exclusion criteria encompassed: (1) non-English publications; (2) review articles and conference abstracts; (3) studies utilizing animal models; (4) publications with a high risk of bias or unavailable full texts; and (5) Studies involving outcome measures that are incapable of being quantified using mean and standard deviation values.

### 2.4 Data extraction

Two authors (Y.Y. and C.S.) independently extracted data: (1) characteristics of the included studies (first author’s surname, publication year, sample size); (2) intervention details (type of intervention, duration, frequency, and session length); (3) participant characteristics (age, BMI, health status); and (4) treatment effects.

### 2.5 Methodological quality assessment

The methodological quality of included studies was evaluated using the Cochrane Risk of Bias tool, which covers seven specific domains: random sequence generation, allocation concealment, blinding of participants and personnel, blinding of outcome assessment, incomplete outcome data, selective reporting, and other potential sources of bias. Each domain was rated as “low risk,” “unclear risk,” or “high risk” according to predefined signaling questions, enabling an overall judgment of the risk of bias for each study (18, 19). Two reviewers (Y.Y. and C.S.) independently performed the quality assessment. Any discrepancies between their evaluations were resolved through consultation with a third reviewer (H.X.) until a consensus was reached.

### 2.6 Certainty of evidence

The certainty of evidence for the effects of isometric exercise training on resting blood pressure was evaluated using the GRADE framework, which classifies evidence as high, moderate, low, or very low. As all analyses were based on randomized controlled trials, the initial certainty of evidence was rated as ‘high’. This rating was subsequently modified based on assessments across five key domains. Two reviewers (Y.Y. and C.S.) independently performed the evaluations, with any discrepancies resolved through discussion or consultation with a third reviewer (H.X.).

The specific assessment criteria are as follows: (1) Risk of Bias: Evidence was downgraded by one level if there were some concerns about bias, and by two levels if the risk of bias was classified as high. (2) Inconsistency: The impact of statistical heterogeneity (*I*^2^) was considered. Evidence was downgraded by one level if the *I*^2^ value was moderate (*I*^2^ > 25%), and by two levels if the *I*^2^ value was high (*I*^2^ > 75%). (3) Indirectness: Evidence was downgraded by one or more levels if there were important differences (indirectness) between the Population, Intervention, Comparator, or Outcomes (PICO) of the included studies and those specified in our review question. (4) Imprecision: Evidence was downgraded for imprecision if the 95% confidence interval around the pooled estimate was wide and crossed a clinically important threshold, or if the total sample size was considered insufficient.

### 2.7 Statistical analysis

Given that systolic and diastolic blood pressure outcomes are consistently measured in standardized units, mean differences were selected as the primary effect measure for data synthesis. Mean differences and their standard deviations were extracted from individual studies to estimate the pooled effect size of isometric exercise training. The *I*^2^ statistic served as the primary indicator of heterogeneity magnitude, with prediction intervals calculated to better represent potential effect variability in future comparable studies.

When significant heterogeneity was identified, defined as an *I*^2^ value exceeding 50%, subgroup and sensitivity analyses were implemented to investigate potential sources of variation. Subgroup analyses and meta-regression were conducted to examine the influence of participant profiles containing health status, and blood pressure level, as well as intervention protocol elements such as exercise type, session frequency, and program duration.

Statistical analyses including Egger’s test, sensitivity analysis, and funnel plot examination were performed using Stata version 18. A *p*-value under 0.05 was seen as statistically significant.

## 3 Results

### 3.1 Studies selection

**Figure 1:**
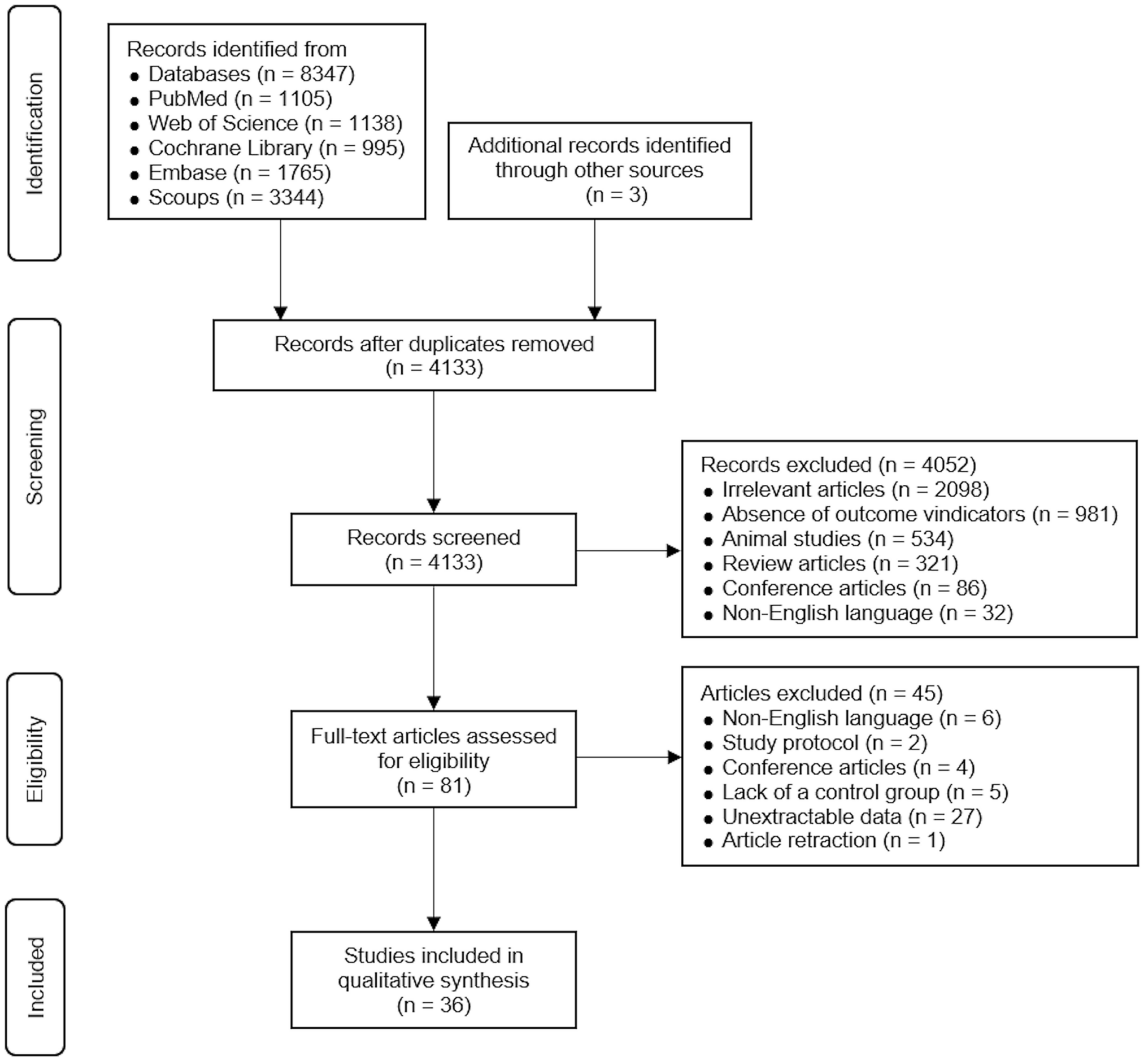
PRISMA flowchart of study selection.

As illustrated in Figure 1, a total of 8,347 articles were initially retrieved from databases, with an additional 3 articles identified from other sources. After removing duplicates, a total of 4,133 studies were retained. After screening the titles and abstracts, 4,052 studies were eliminated since they failed to satisfy the pre-established inclusion criteria. Subsequently, the full-text versions of 81 studies were evaluated for eligibility. Among these, 45 studies were excluded, and the reasons are as follows: (1) non-English publications (*n* = 6); (2) study protocols (*n* = 2); (3) conference articles (*n* = 4); (4) absence of a control group (*n* = 5); (5) inability to extract data (*n* = 27); and (6) retracted articles (*n* = 1). Ultimately, 36 studies (10–12, 20–52) were included.

### 3.2 Characteristics of the included studies

The main characteristics of the included studies are summarized in Table 1. A total of 36 studies (encompassing 40 randomized controlled trials) were included, published between 2002 (20) and 2025 (21) and spanning multiple countries, including Australia (10, 22), Brazil (23–26), Canada (27), Colombia (11), Denmark (12), Germany (28), India (29, 30), Japan (21, 31, 32), Portugal (33), South Africa (34), the UK (35–47), and the USA (20, 48–50). The intervention and control groups comprised 844 and 849 participants, respectively. In individual studies, the number of participants ranged from 7 (36) to 200 (49), with ages spanning from 18 (46) to 71 (33) years. Both single-gender and mixed-gender reports were included. The participant groups were characterized as follows: healthy (12, 22, 24, 27, 34, 36–38, 41, 46, 50) (188 individual), sedentary (20, 35, 39, 44, 45) (68 individuals), hypertensive (10, 11, 25, 26, 28, 30–33, 40, 42, 48, 51, 52) (278 individuals), pre-hypertensive (21, 29, 43, 47, 49) (281 individuals), and those with peripheral artery disease (23) (102 individuals). Among the 36 studies (encompassing 40 randomized controlled trials), all 40 RCTs reported data for systolic blood pressure, and 39 RCTs reported data for diastolic blood pressure the exercise modalities across these RCTs were as follows: 25 RCTs employed isometric handgrip training (IHG) (10–12, 21–34, 36, 44, 48–51), 8 RCTs employed isometric wall squat training (IWST) (11, 35, 40–43, 45, 47), 6 RCTs employed bilateral isometric leg training (BILT) (20, 38, 39, 46), and 1 RCT employed isometric training (IIT) (37). The intervention durations varied, ranging from a minimum of 1 week (34) to a maximum of 12 months (43), with frequencies ranging from a minimum of 3 (36) sessions per week to a maximum of 7 (49) sessions per week. The intervention intensity ranged from a low of 10% MVC (22) to a high of 95% HR peak (35). Additionally, we calculated the weekly exercise time based on frequency and intervention duration, with the time range spanning from 9 min (33) to 24 min (39).

**Table 1:** Characteristics of the studies included in this meta-analysis.

Author/year	Country	Sample size (*n*)	Gender	Participants	Mean age (y)	Type of intervention	Frequency (times/week)	Duration (weeks)	Exercise intensity	Supervision	Outcomes
Baddeley-White et al., 2019 (36)	UK	Con: 8 Int: 7	5/3 4/3	Healthy adults	26.0 ± 5.1 28.8 ± 5.9	Non-Intervention IHG	3	4	30% MVC	Yes	SBP, DBP
Baddeley-White et al., 2021 (37)	UK	Con: 10 Int:10	6/4 5/5	Healthy adults	30.7 ± 8.8 29.3 ± 7.9	Non-Intervention IIT	3	6	30% MVC	Yes	SBP, DBP
Badrov et al., 2013 (52)	Canada	Con: 12 Int: 12	7/5 6/6	Hypertensives patients	63 ± 9 65 ± 7	Non-Intervention IHG	3	10	30% MVC	Yes	SBP, DBP
Badrov et al., 2013 (27)	Canada	Con: 9 Int: 12	0/9 0/12	Healthy adults	24 ± 8 23 ± 4	Non-Intervention IHG	3	8	30% MVC	Yes	SBP, DBP
Badrov et al., 2013 (27)	Canada	Con: 9 Int: 11	0/9 0/11	Healthy adults	24 ± 8 27 ± 6	Non-Intervention IHG	5	8	30% MVC	Yes	SBP, DBP
Baross et al., 2012 (39)	UK	Con: 10 Int: 10	10/0 10/0	Sedentary individuals	53.4 ± 5.0 53.6 ± 5.5	Non-Intervention BILT	3	8	70%HR peak	Yes	SBP, DBP
Baross et al., 2012 (39)	UK	Con: 10 Int: 10	10/0 10/0	Sedentary individuals	53.4 ± 5.0 54.6 ± 5.5	Non-Intervention BILT	3	8	85%HR peak	Yes	SBP, DBP
Baross et al., 2013 (38)	UK	Con: 10 Int: 10	10/0 10/0	Healthy adults	53 ± 5 53 ± 6	Non-Intervention BILT	3	8	85% HR peak	Yes	SBP, DBP
Baross et al., 2017 (44)	UK	Con: 12 Int: 12	7/5 6/6	Sedentary individuals	21.3 ± 2.0 20.7 ± 1.6	Non-Intervention IHG	4	6	20% MVC	Yes	SBP, DBP
Carlson et al., 2016 (10)	Australian	Con: 20 Int: 20	8/12 7/13	Hypertensive patients	54 ± 8 52 ± 8	Non-Intervention IHG	3	8	30% MVC	Yes	SBP, DBP
Cohen et al., 2023 (11)	Colombia	Con: 22 Int: 28	n/a n/a	Hypertensive patients	44.9 ± 9.6 44.9 ± 9.6	Healthy lifestyles IHG	3	12	30% MVC	Yes	SBP, DBP
Cohen et al., 2023 (11)	Colombia	Con: 22 Int: 27	n/a n/a	Hypertensive patients	44.9 ± 9.6 44.9 ± 9.6	Healthy lifestyles IWST	3	12	30% MVC	Yes	SBP, DBP
Correia et al., 2020 (23)	Brazil	Con: 52 Int: 50	32/20 33/17	PAD	67 ± 11 66 ± 12	Non-Intervention IHG	3	8	30% MVC	No	SBP, DBP
Danielsen et al., 2024 (12)	Denmark	Con: 24 Int: 24	15/9 15/9	Healthy adults	62.7 ± 9.2 65.2 ± 7.2	Usual care IHG	3	20	30%MVC	No	SBP, DBP
Decaux et al., 2022 (35)	UK	Con: 10 Int: 10	n/a n/a	Sedentary individuals	28.3 ± 5.6 31.4 ± 6	Non-Intervention IWST	3	4	95% HR peak	Yes	SBP, DBP
Edwards et al., 2023 (45)	UK	Con: 17 Int: 17	17/0 17/0	Sedentary individuals	45 ± 5.9 45 ± 5.9	Non-Intervention IWST	3	4	95% HR peak	Yes	SBP, DBP
Farah et al., 2018 (24)	Brazil	Con: 16 Int: 14	n/a n/a	Healthy adults	58 ± 3 59 ± 2	Non-Intervention IHG	3	12	30%MVC	Yes	SBP, DBP
Gordon et al., 2018 (48)	USA	Con: 5 Int: 8	2/3 2/6	Hypertensive patients	47 ± 9 53 ± 5	Non-Intervention IHG	3	12	30% MVC	Yes	SBP, DBP
Hess et al., 2016 (22)	Australian	Con: 10 Int: 10	6/4 6/4	Healthy adults	38.8 ± 10.5 38.7 ± 12.6	Non-InterventionIHG	3	6	10% MVC	Yes	SBP, DBP
Howden et al., 2002 (20)	USA	Con: 16 Int: 9	11/5 7/2	Sedentary individuals	24.5 ± 6.1 21.1 ± 1.2	Non-Intervention BILT	3	5	20% MVC	Yes	SBP, DBP
Lea et al., 2024 (47)	UK	Con: 10 Int: 10	n/a n/a	Pre-hypertensive patients	28 ± 4 39 ± 15	Non-Intervention IWST	3	4	95% HR peak	No	SBP, DBP
Mortimer et al., 2011 (34)	South African	Con: 9 Int: 9	0/9 0/9	Healthy adults	49.88 ± 1.4 47.88 ± 1.8	Non-Intervention IHG	5	1	30%MVC	Yes	SBP, DBP
Nemoto et al., 2021 (32)	Japan	Con: 26 Int: 27	16/10 14/13	Hypertensive patients	61.2 ± 13.3 62.3 ± 11.7	Non-Intervention IHG	3	8	30% MVC	No	SBP, DBP
Nemoto et al., 2025 (21)	Japan	Con: 27 Int: 29	13/14 12/17	Pre-hypertensive patients	65.7 ± 11.8 68.1 ± 10.9	Non-Intervention IHG	3	12	15%MVC	Yes	SBP, DBP
O’Driscoll et al., 2022 (43)	UK	Con: 12 Int: 12	12/0 12/0	Pre-hypertensive patients	41.3–49 41.3–49	Non-Intervention IWST	3	52	95% HR peak	No	SBP, DBP
Ogbutor et al., 2019 (49)	USA	Con: 200 Int: 200	112/88 109/91	Pre-hypertensive patients	41.27 ± 6.31 40.78 ± 6.04	Non-Intervention IHG	7	4	30%MVC	Yes	SBP, DBP
Okamoto et al., 2020 (31)	Japan	Con: 11 Int: 11	4/7 5/6	Hypertensive patients	64 ± 11 65 ± 11	Non-Intervention IHG	5	8	30% MVC	No	SBP, DBP
Pagonas et al., 2017 (28)	Germany	Con: 25 Int: 25	11/14 9/16	Hypertensive patients	62.1 ± 7.1 58.8 ± 10.6	Non-Intervention IHG	5	12	30% MVC	Yes	SBP, DBP
Palmeira et al., 2021 (25)	Brazil	Con: 32 Int: 31	31/1 27/4	Hypertensive patients	52.7 ± 2.6 54.3 ± 3.7	Anti-hypertensive medications IHG	3	12	30% MVC	Yes	SBP, DBP
Pinto et al., 2024 (33)	Portugal	Con: 23 Int: 27	10/13 12/15	Hypertensive patients	71.0 ± 3.9 71.6 ± 3.6	Usual care IHG	3	8	50%MVC	No	SBP, DBP
Punia et al., 2020 (30)	India	Con: 20 Int: 20	n/a n/a	Hypertensive patients	30–45 30–45	Non-Intervention IHG	3	8	30% MVC	No	SBP, DBP
Cahu Rodrigues et al., 2020 (26)	Brazil	Con: 16 Int: 17	5/11 6/11	Hypertensive patients	59 ± 2 61 ± 2	Non-Intervention IHG	3	12	30% MVC	Yes	SBP
Spitz et al., 2024 (50)	USA	Con: 44 Int: 47	19/25 21/26 18/23	Healthy adults	21 21	Non-Intervention IHG	3	6	30% MVC	Yes	SBP, DBP
Sultana et al., 2024 (29)	India	Con: 30 Int: 30	30/0 30/0	Pre-hypertensive patients	/	Non-Intervention IHG	3	12	30%MVC	Yes	SBP, DBP
Taylor et al., 2003 (51)	Canada	Con: 8 Int: 9	5/3 5/4	Hypertensive patients	64.2 ± 5.5 69.3 ± 6.0	Non-Intervention IHG	3	10	30% MVC	Yes	SBP, DBP
Taylor et al., 2019 (42)	UK	Con: 24 Int: 24	n/a n/a	Hypertensive patients	43.8 ± 7.3 43.8 ± 7.3	Non-Intervention IWST	3	4	30% MVC	Yes	SBP, DBP
Wiles et al., 2010 (46)	UK	Con: 11 Int: 11	11/0 11/0	Healthy adults	18–34 18–34	Non-Intervention BILT	3	8	70% HR peak	Yes	SBP, DBP
Wiles et al., 2010 (46)	UK	Con: 11 Int: 11	11/0 11/0	Healthy adults	18–34 18–34	Non-Intervention BILT	3	8	85% HR peak	Yes	SBP, DBP
Wiles et al., 2017 (41)	UK	Con: 12 Int: 12	12/0 12/0	Healthy adults	30 ± 7 30 ± 7	Non-Intervention IWST	3	4	95% HR peak	No	SBP, DBP
Wiles et al., 2024 (40)	UK	Con: 19 Int: 22	8/11 9/13	Hypertensive patients	56.2 ± 14.3 57.0 ± 15.2	Non-Intervention IWST	3	21	95% HR peak	No	SBP, DBP

IHG, Isometric Handgrip Training; IWST, Isometric Wall Squat Training; BILT, Bilateral Isometric Leg Training; MVC, Maximal Voluntary Contraction; HR peak, Peak Heart Rate; SBP, Systolic blood pressure; DBP, Diastolic blood pressure.

### 3.3 Main effect

**Figure 2:**
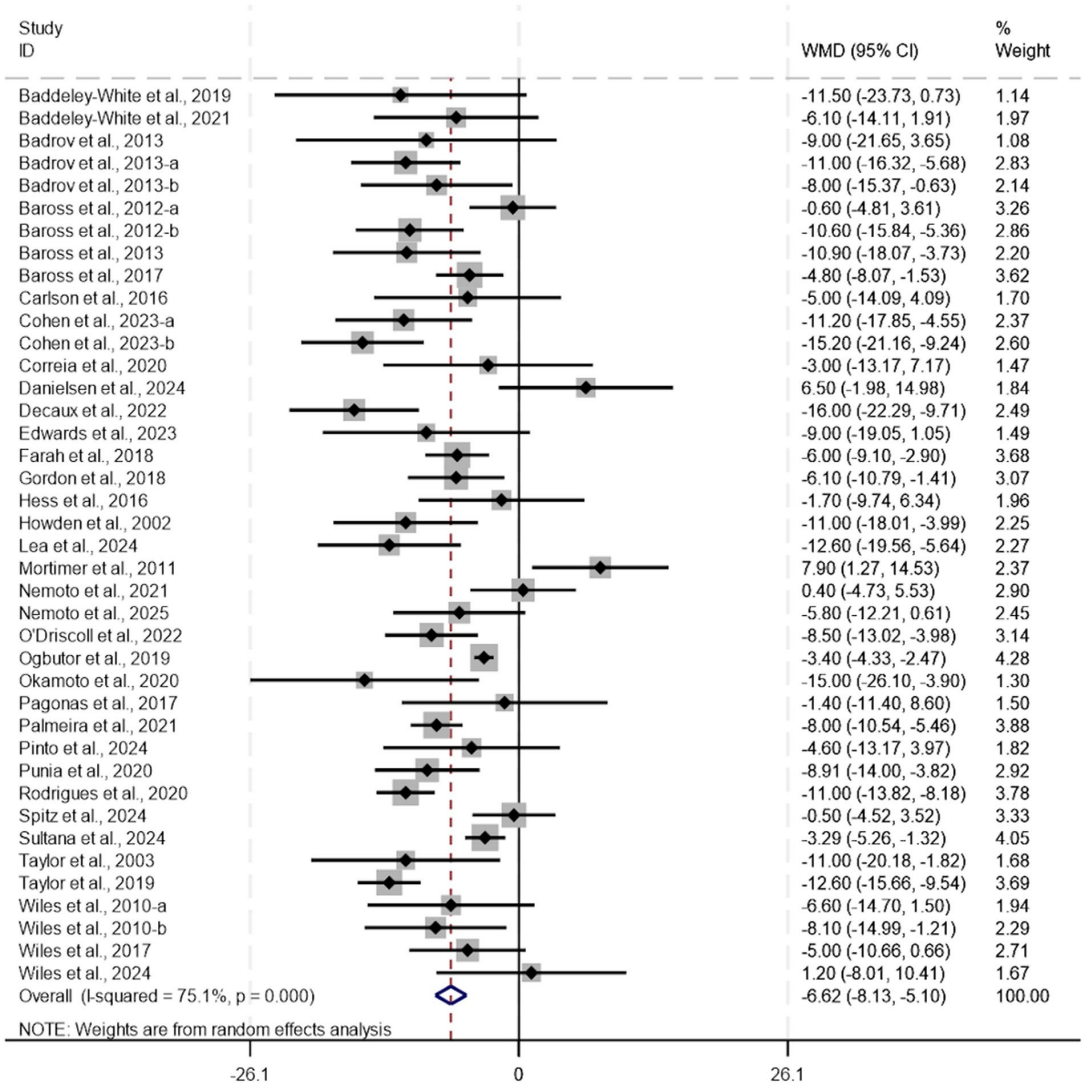
A meta-analysis of the effects of isometric training on systolic blood pressure.

**Figure 3:**
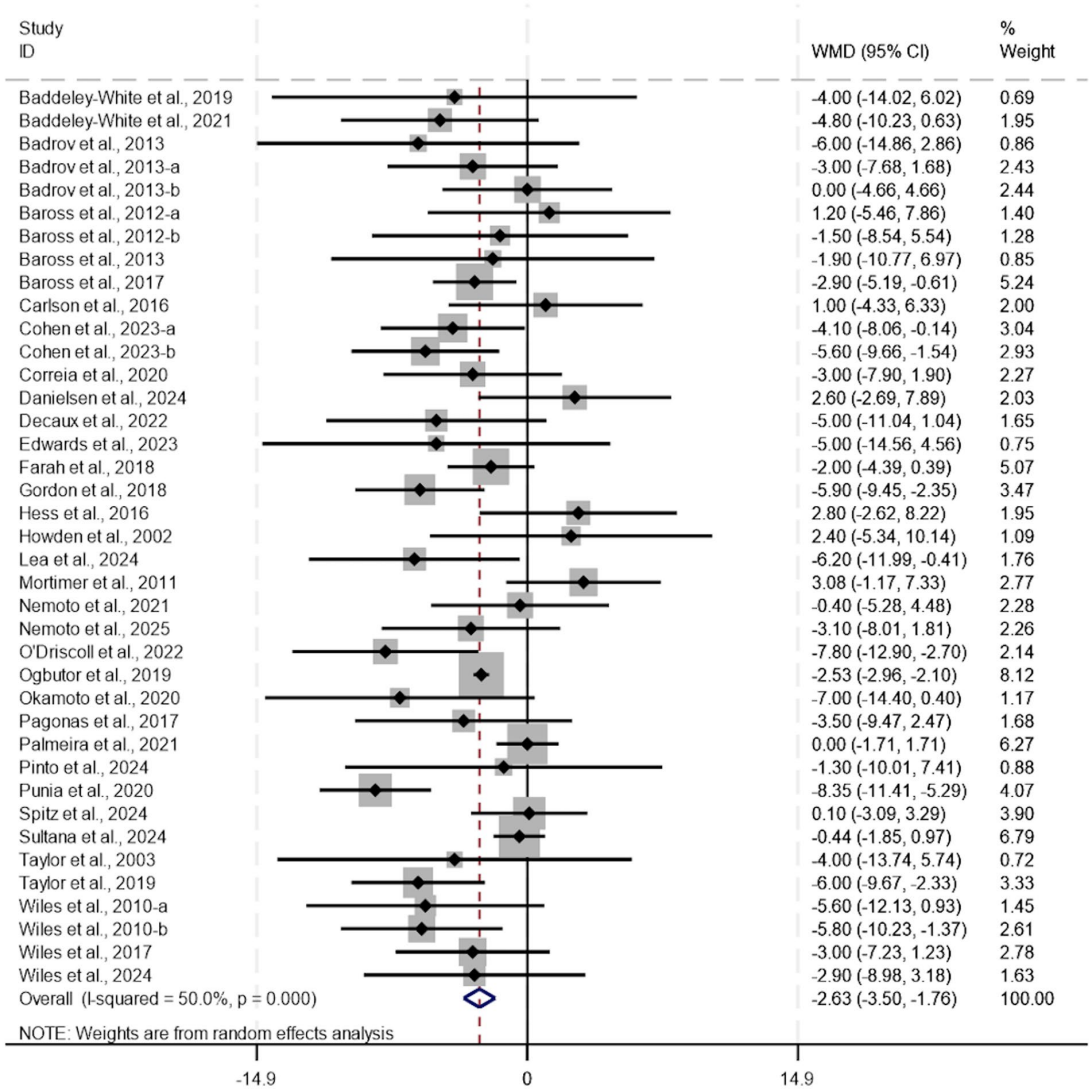
A meta-analysis of the effects of isometric training on diastolic blood pressure.

Isometric training can effectively reduce systolic blood pressure. (WMD, −6.62; 95% CI, −8.13 to −5.10, *p* < 0.0001, *I*^2^ = 75%, Figure 2) and diastolic blood pressure (WMD, −2.63; 95% CI, −3.50 to −1.76, *p* < 0.0001, *I*^2^ = 50%, Figure 3). Meta-analysis indicated substantial heterogeneity in both systolic and diastolic blood pressure outcomes. To explore potential sources of this variability and identify modifiable exercise-related factors, additional analyses including meta-regression, subgroup analysis, and sensitivity analysis were performed. It should be noted that the following subgroup analyses are exploratory in nature, aimed at generating hypotheses, and their findings are susceptible to ecological bias. They should not be interpreted as definitive evidence of causal relationships.

### 3.4 Meta-regression

**Figure 4:**
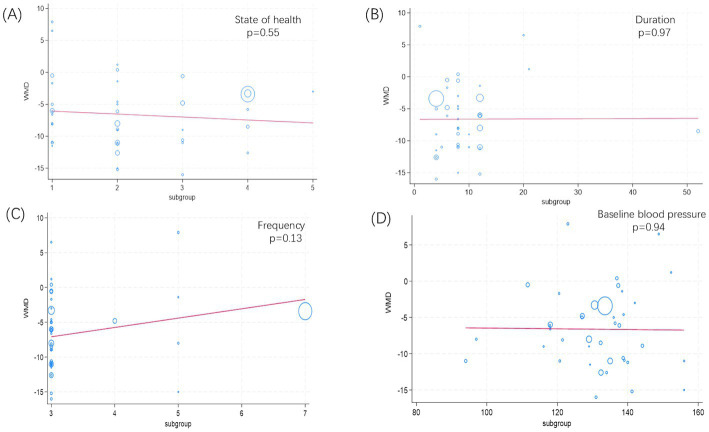
Meta-regression analysis results for isometric exercise training on SBP. **(A)** State of health; **(B)** Duration; **(C)** Frequency; **(D)** Baseline blood pressure.

As shown in Figure 4, the results of the meta-regression suggest no significant association between health status (*p* = 0.55, Figure 4A), duration (*p* = 0.97, Figure 4B), frequency (*p* = 0.13, Figure 4C), baseline blood pressure (*p* = 0.94, Figure 4D), and the reduction in systolic blood pressure achieved through isometric training.

**Figure 5:**
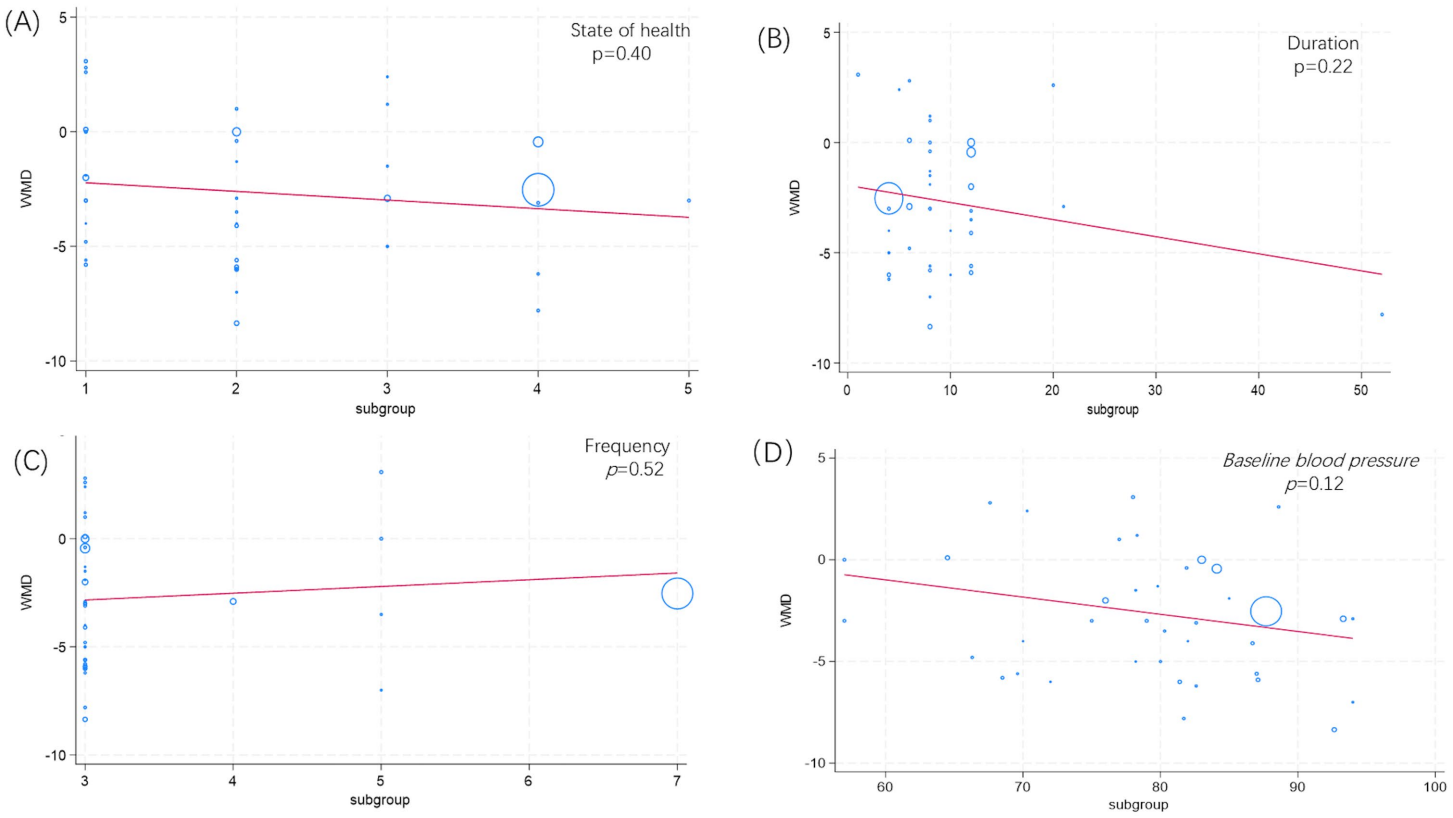
Meta-regression analysis results for isometric exercise training on DBP. **(A)** State of health; **(B)** Duration; **(C)** Frequency; **(D)** Baseline blood pressure.

As shown in Figure 5, meta-regression analysis indicates no significant association between health status (*p* = 0.40, Figure 5A), duration (*p* = 0.22, Figure 5B), frequency (*p* = 0.52, Figure 5C), baseline blood pressure (*p* = 0.12, Figure 5D), and the reduction in diastolic blood pressure achieved through isometric training.

### 3.5 Subgroup analysis

#### 3.5.1 Systolic blood pressure

The results of subgroup analyses are presented in Table 2. The blood pressure-lowering effects of isometric exercise training were significantly influenced by factors including sex, health status, baseline blood pressure levels, as well as exercise modality, frequency, intensity, and duration.

**Table 2:** Subgroup analysis results for systolic blood pressure (random−effects model).

Subgroup	*K* (*n*)	MD (95% CI) mmHg	*p*_d_	*p*_m_	*I* ^2^
Gender				*p* < 0.0001	71%
Male	9 (123)	−6.23 [−8.81, −3.66]	*p* < 0.00001		55%
Female	3 (32)	−5.91 [−8.76, −3.06]	*p* = 0.53		90%
Health status				*p* < 0.00001	76%
Healthy adults	13 (183)	−4.57 [−7.66, −1.48]	*p* = 0.0002		68%
Sedentary	6 (68)	−8.22 [−12.80, −3.63]	*p* = 0.0004		77%
Pre−hypertensive	5 (281)	−5.14 [−7.41, −2.87]	*p* < 0.00001		65%
Hypertensive	15 (278)	−8.22 [−10.57, −5.86]	*p* < 0.00001		62%
Baseline blood pressure				*p* < 0.00001	75%
<120	7 (117)	−6.16 [−9.14, −3.18]	*p* < 0.00001		71%
≥120–140	25 (561)	−6.45 [−8.30, −4.60]	*p* < 0.00001		79%
≥140	8 (161)	−7.32 [−12.46, −2.18]	*p* = 0.0009		60%
Form of exercise				*p* < 0.00001	76%
BILT	6 (61)	−7.63 [−11.74, −3.52]	*p* = 0.0003		62%
IWST	8 (125)	−10.29 [−13.60, −6.98]	*p* < 0.00001		61%
IHG	25 (643)	−5.24 [−6.93, −3.55]	*p* < 0.00001		72%
Frequency (per week)				*p* < 0.00001	71%
3	34 (572)	−7.19 [−8.85, −5.53]	*p* < 0.00001		69%
5	4 (55)	−3.66 [−13.61, 6.29]	*p* = 0.47		82%
Duration				*p* < 0.00001	75%
≤8	26 (582)	−6.55 [−8.59, −4.51]	*p* < 0.00001		76%
>8	14 (257)	−6.82 [−9.20, −4.44]	*p* < 0.0001		72%
Intensity				*p* < 0.00001	77%
20%MVC	2 (21)	−7.09 [−12.96, −1.23]	*p* = 0.02		59%
30%MVC	24 (630)	−6.26 [−8.27, −4.26]	*p* < 0.00001		81%
70%HR peak	2 (21)	−2.56 [−8.08, 2.95]	*p* = 0.36		40%
85%HR peak	3 (31)	−9.99 [−13.60, −6.38]	*p* < 0.00001		0%
95%HR peak	6 (74)	−8.69 [−13.02, −4.37]	*p* < 0.0001		60%

*K* (*n*), the number of studies included in the pooled effect analysis (total number of participants across pooled studies); *p*_d_, the *p*−value used to assess differences in effect size between subgroups; *p*_m_, the *p*−value for the heterogeneity test.

Specifically, the largest reductions in blood pressure were observed in males (WMD, −6.23; 95% CI, −8.81 to −3.66, *p* < 0.00001, *I*^2^ = 55%), hypertensive individuals (WMD, −8.22; 95% CI, −10.57 to −5.86, *p* < 0.00001, *I*^2^ = 62%), and those with baseline systolic blood pressure ≥140 mmHg (WMD, −8.08; 95% CI, −12.42 to −3.74, *p* = 0.0003, *I*^2^ = 60%).

Furthermore, the greatest blood pressure reduction was associated with isometric exercise regimens that involved wall squat training (WMD, −10.29; 95% CI, −13.60 to −6.98, *p* < 0.00001, *I*^2^ = 61%), three sessions weekly (WMD: −7.19; 95% CI, −8.85 to −5.53, *p* < 0.00001, *I*^2^ = 69%), an intensity of 85% HR peak (WMD, −9.99; 95% CI, −13.60 to −6.38, *p* < 0.00001, *I*^2^ = 0%), and a duration of more than 8 weeks (WMD, −6.82; 95% CI, −9.20 to −4.44, *p* < 0.0001, *I*^2^ = 72%).

#### 3.5.2 Diastolic blood pressure

The results of subgroup analyses are summarized in Table 3. The antihypertensive effect of isometric exercise training was significantly influenced by sex, health status, baseline blood pressure levels, exercise modality, frequency, intensity, and intervention duration (*p* < 0.05).

**Table 3:** Subgroup analysis results for diastolic blood pressure (random-effects model).

Subgroup	*K* (*n*)	MD (95% CI) mmHg	*p_d_*	*p_m_*	*I^2^*
Gender				*p* = 0.02	45%
Male	9 (123)	−3.07 [−5.33, −0.82]	*p* = 0.008		46%
Female	3 (32)	0.14 [−3.35, 3.63]	*p* = 0.94		44%
Health status				*p* = 0.002	51%
Healthy adults	13 (183)	−1.40 [−3.01, 0.21]	*p* = 0.11		35%
Sedentary	6 (68)	−2.45 [−4.32, −0.59]	*p* = 0.01		0%
Pre-hypertensive	5 (281)	−2.74 [−4.60, −0.88]	*p* = 0.004		71%
Hypertensive	14 (262)	−3.87 [−5.88, −1.87]	*p* = 0.0002		64%
Baseline blood pressure				*p* = 0.0002	50%
<80	19 (285)	−1.51 [−2.75, −0.28]	*p* = 0.02		12%
≥80–89	16 (482)	2.60 [−2.69, 7.89]	*p* = 0.001		59%
≥90	4 (56)	−5.22 [−8.64, −1.79]	*p* = 0.003		65%
Form of exercise				*p* = 0.0002	51%
BILT	6 (61)	−2.65 [−5.50, 0.21]	*p* = 0.07		13%
IWST	8 (125)	−5.26 [−7.02, −3.51]	*p* < 0.00001		0%
IHG	24 (627)	−1.96 [−3.00, −0.93]	*p* = 0.0002		59%
Frequency (per week)				*p* = 0.0002	51%
3	33 (572)	−2.89 [−4.02, −1.76]	*p* = 0.0004		52%
5	5 (67)	−1.58 [−4.54, 1.38]	*p* = 0.30		54%
Duration				*p* = 0.0002	50%
≤8	26 (583)	−2.67 [−3.80, −1.55]	*p* < 0.00001		44%
>8	13 (240)	−2.70 [−4.25, −1.16]	*p* = 0.007		56%
Intensity				*p* = 0.0002	52%
20%MVC	2 (21)	−1.59 [−6.07, 2.88]	*p* = 0.49		40%
30%MVC	23 (613)	−2.45 [−3.54, −1.35]	*p* < 0.0001		63%
70%HR peak	2 (21)	−2.23 [−8.90, 4.43]	*p* = 0.51		51%
85%HR peak	3 (31)	−4.17 [−7.63, −0.72]	*p* = 0.02		0%
95%HR peak	6 (74)	−4.87 [−7.17, −2.57]	*p* < 0.0001		0%

*K* (*n*), the number of studies included in the pooled effect analysis (total number of participants across pooled studies); *p*_d_, the *p*-value used to assess differences in effect size between subgroupps; *p*_m_, the *p*-value for the heterogeneity test.

Specifically, largest reductions in blood pressure were observed in males (WMD, −3.07; 95% CI, −5.33 to −0.82, *p* = 0.008, *I*^2^ = 46%), hypertensive participants (WMD, −3.87; 95% CI, −5.88 to −1.87, *p* = 0.0002, *I*^2^ = 64%), and those with baseline diastolic blood pressure ≥90 mmHg (WMD, −5.22; 95% CI, −8.64 to −1.79, *p* = 0.003, *I*^2^ = 65%).

Furthermore, the greatest blood pressure-lowering outcomes were associated with the following exercise regimens: wall squat training (WMD, −5.26; 95% CI, −7.02 to −3.51, *p* < 0.00001, *I*^2^ = 0%), three sessions weekly (WMD, −2.89; 95% CI, −4.02 to −1.76, *p* = 0.0004, *I*^2^ = 52%), an intensity of 95% HR peak (WMD, −4.87; 95% CI, −7.17 to −2.57, *p* < 0.0001, *I*^2^ = 0%), and an intervention duration exceeding 8 weeks (WMD, −2.70; 95% CI, −4.25 to −1.16, *p* = 0.007, *I*^2^ = 56%).

### 3.6 Risk of bias

**Figure 6:**
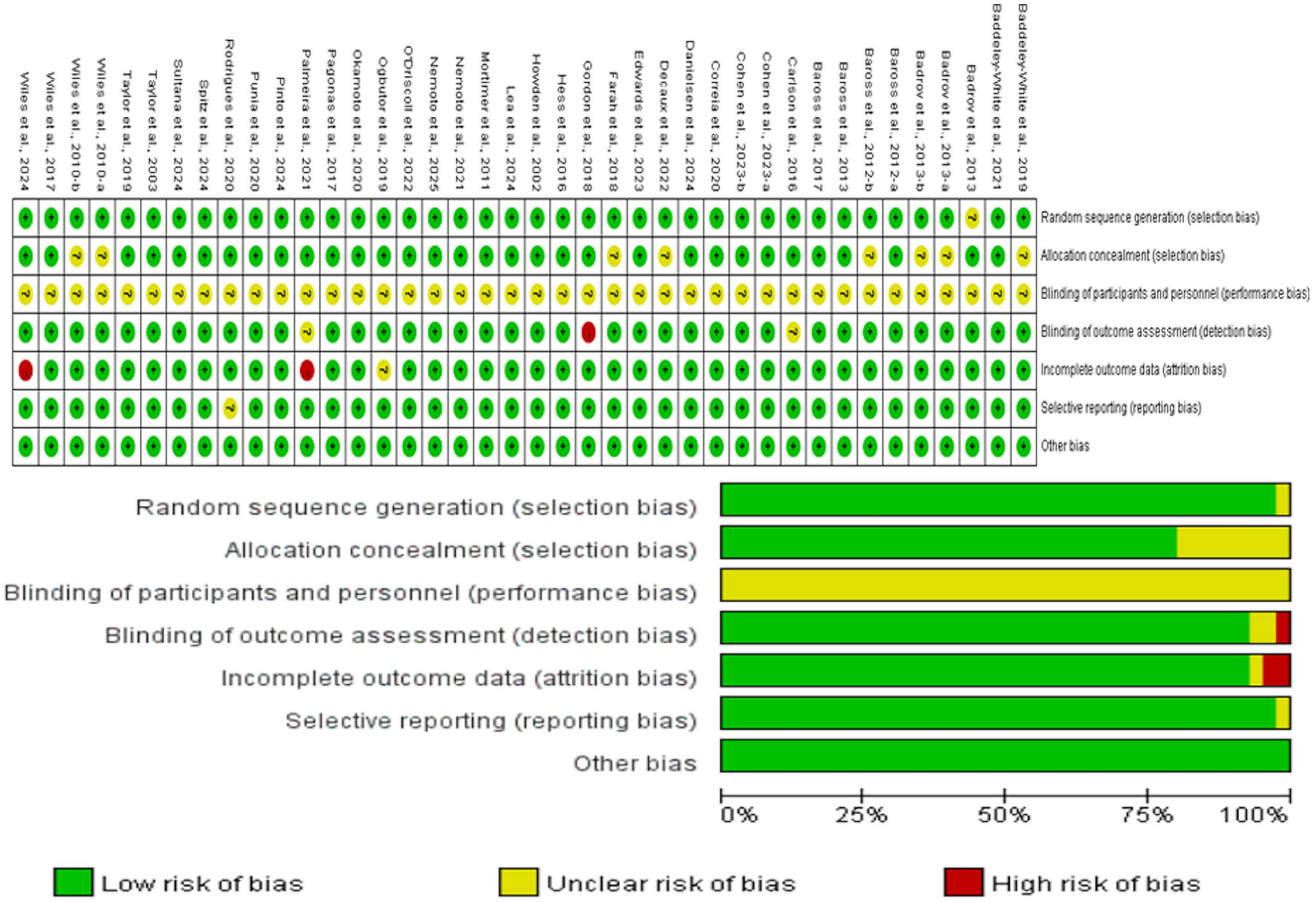
Risk assessment of included studies.

The methodological quality of the included randomized controlled trials (RCTs) was evaluated using the Cochrane Risk of Bias 2 (RoB 2) tool (Figure 6). A common and inherent methodological constraint in exercise intervention trials is the difficulty of blinding participants and personnel, resulting in a prevalent high or unclear risk of performance bias across most studies. Notwithstanding this limitation, the risks of bias in other key domains were generally low. The majority of trials reported adequate methods for random sequence generation, allocation concealment, and blinding of outcome assessment. Furthermore, incomplete outcome data and potential selective reporting were appropriately addressed in most cases. Overall, while the inherent risk of performance bias must be considered, the included studies demonstrated sound methodological rigor in trial design and outcome measurement within this field, thereby supporting the reliability of the synthesized evidence.

### 3.7 GRADE evaluation of evidence quality

Using the GRADE framework, the certainty of evidence was evaluated for the effect of isometric exercise training on resting blood pressure. The overall rating for both SBP and DBP outcomes was moderate (Table 4). All data were derived from randomized controlled trials, this rating began as ‘high’ and was downgraded by one level due to inconsistency, attributable to considerable heterogeneity in the pooled estimates (*I^2^* = 74% for SBP; *I^2^* = 48% for DBP).

**Table 4:** GRADE assessment of primary outcomes.

Outcome	No of participants (studies)	Certainty assessment					WMD (95% CI)	GRADE*
		Risk of bias	Inconsistency	Indirectness	Imprecision	Other considerations		
SBP	1,693 (40 RCTs)	Not serious	Serious	Not serious	Not serious	Not serious	−6.72 [−8.21, −5.23]	⊕ ⊕ ⊕ ◯ Moderate
DBP	1,657 (39 RCTs)	Not serious	Serious	Not serious	Not serious	Not serious	−2.72 [−3.57, −1.87]	⊕ ⊕ ⊕ ◯ Moderate

### 3.8 Publication bias

**Figure 7:**
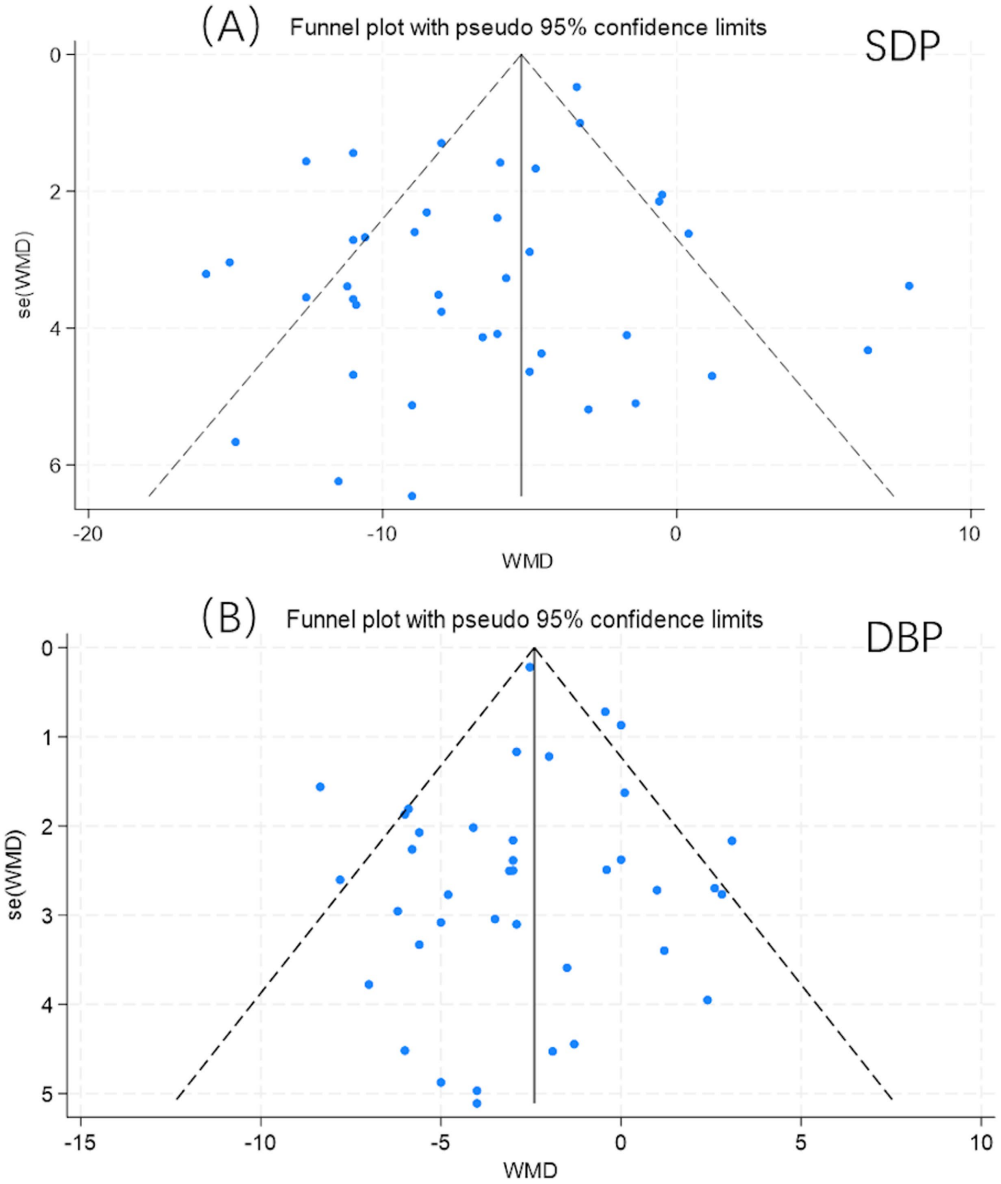
Results of funnel plot. **(A)** Results of funnel plot on SBP; **(B)** Results of funnel plot on DBP.

Funnel plots were employed to analyses publication bias. Visual inspection of the funnel plot (Figure 7A) combined with the Egger test (*t* = −2.07, *p* = 0.045, Table 5). This suggests the potential presence of publication bias or small-study effects, where smaller studies showing larger effect sizes might be more likely to be published.

**Table 5:** Results of Egger’s test (SBP).

Std_eff	Coefficient	Std. err.	*t*	*p* > |*t*|	[95% conf., interval]
Slope	−3.796443	0.9294134	−4.08	0.000	(−5.677942, −1.914944)
Bias	−0.9941771	0.4792466	−2.07	0.045	(−1.964361, −0.023993)

Std. err, standard error; *t*, *t*-test statistic; *p*, probability.

To estimate and adjust for the potential impact of any missing studies, we applied the Trim and Fill method. The raw pooled effect size for isometric exercise on systolic blood pressure was −6.62 mmHg, and it remained unchanged after adjustment (−6.62 mmHg, 95% CI: −8.131 to −5.099, *p* < 0.0001). This analysis indicated that no studies needed to be imputed to achieve symmetry in the funnel plot. Thus, the pooled effect estimate for systolic blood pressure reduction was robust to this adjustment. While the Egger’s test indicates asymmetry, the lack of imputation by the Trim and Fill procedure suggests that any potential publication bias may not be substantial enough to qualitatively alter the main conclusion that isometric exercise significantly reduces systolic blood pressure. Nevertheless, the possibility of a modest overestimation of the true effect size due to small-study effects cannot be ruled out.

Similarly, funnel plots were employed to detect publication bias across all 39 trials for diastolic blood pressure. Visual inspection of the funnel plots (Figure 7B) and the Egger test (*t* = −0.52, *p* = 0.606, Table 6) indicated no evidence of publication bias, rendering the results suitable for meta-analysis.

**Table 6:** Results of Egger’s test (DBP).

Std_eff	Coefficient	Std. err.	*t*	*p* > |*t*|	[95% conf., interval]
Slope	−2.301857	0.3278464	−7.02	0.000	(−2.966137, −1.637577)
Bias	−0.1513385	0.2905962	−0.52	0.606	(−0.7401422, 0.4374653)

Std. err, standard error; *t*, *t*-test statistic; *p*, probability.

### 3.9 Sensitivity analyses

**Figure 8:**
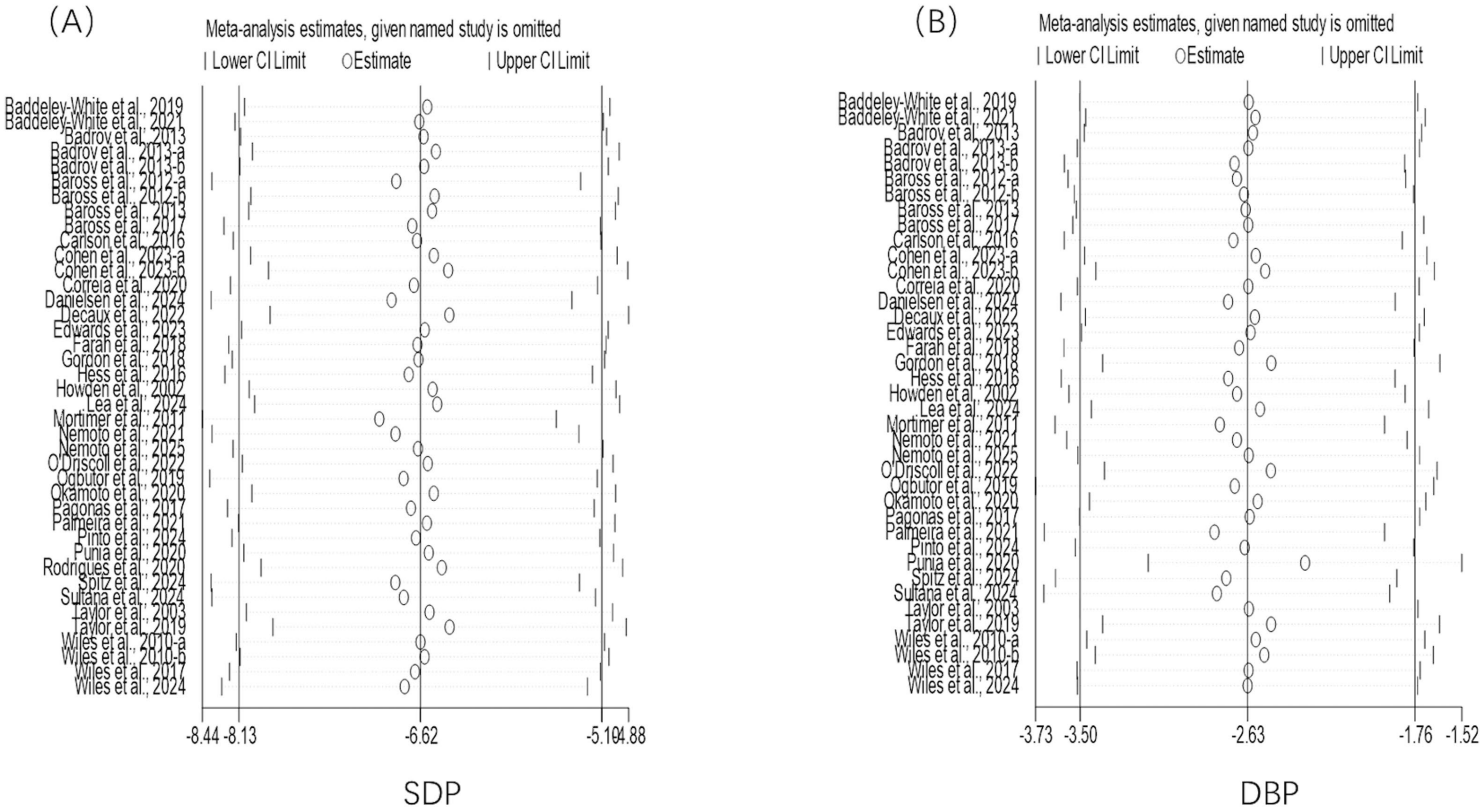
Results of sensitivity analyses. **(A)** Results of sensitivity analyses on SBP; **(B)** Results of sensitivity analyses on SBP.

Sensitivity analysis indicates that excluding any single study does not alter the consistent and stable positive effects of isometric training on systolic blood pressure (Figure 8A) and diastolic blood pressure (Figure 8B), with both the direction and magnitude of these effects remaining unchanged.

## 4 Discussion

### 4.1 Main findings

The study aimed to assess how isometric training affects resting blood pressure and identify key parameters of exercise protocols associated with blood pressure control. From an initial pool of 8,347 records, 36 studies met the eligibility criteria for systematic review and meta-analysis. We examined potential influencing factors derived from participant baseline characteristics and exercise training protocols.

Meta-analysis revealed that isometric exercise significantly reduced both systolic and diastolic blood pressure compared with non-intervention controls. While meta-regression did not identify statistically significant moderators, exploratory subgroup analyses suggested that larger reductions in blood pressure were associated with isometric wall squat training conducted three times per week for a duration of at least 8 weeks. Regarding exercise intensity, the reductions appeared most pronounced at higher intensities; however, the specific intensity associated with the greatest effect differed between systolic (85% HR peak) and diastolic (95% HR peak) blood pressure.

### 4.2 Systolic blood pressure

Our results demonstrate that isometric exercise training significantly reduces resting systolic blood pressure with a mean reduction of 6.72 mmHg compared to non-exercise control groups (53). This finding aligns with prior meta-analyses reporting reductions of 5–9 mmHg, reinforcing the potential role of isometric exercise as a non-pharmacological intervention for blood pressure management.

Our study extends the evidence base by including a broader range of randomized controlled trials and participant demographics than earlier reviews with more limited samples (1, 16, 18, 54). Specifically, we incorporated a larger number of trials involving young healthy adults, middle-aged and older individuals, women, and prehypertensive populations. Through subgroup and meta-regression analyses, we further identified several factors that may influence treatment effects, including age, sex, baseline blood pressure levels, and exercise parameters such as modality, intensity, frequency, and duration.

Regarding sex differences, our analysis indicated a larger average reduction in males. Badrov et al. (52) demonstrated that males exhibit more pronounced reductions following isometric handgrip training. A hypothesized mechanism for this difference centers on greater muscle mass, potentially promoted by androgens like testosterone. It is proposed that greater muscle mass and strength, which are promoted by androgens such as testosterone. This could stimulate vascular endothelial cells through mechanical stretch and metabolite accumulation to release vasodilators including nitric oxide, thereby promoting vasodilation and reducing blood pressure (52, 55). However, this remains a plausible hypothesis within a complex adaptive process. Further research is needed to clarify the interactions between sex hormones, exercise modality, and neuro-humoral adaptations.

Concerning health status, our subgroup analysis found the largest average reduction in hypertensive individuals, which aligns with observations by Carlson et al. (10). Furthermore, participants with higher baseline systolic blood pressure (≥140 mmHg) showed a greater average reduction in our analysis, a pattern consistent with findings by Howden et al. (20) who suggested enhanced responsiveness to exercise stimuli in this group. These observations support the notion that the potential benefit of isometric training may be more pronounced in individuals with elevated blood pressure.

Regarding exercise modality differences, Baross et al. (38) demonstrated the superior efficacy of isometric wall squat training, which engages large muscle groups through static contraction. It is hypothesized that this significantly increases venous return resistance in the lower limbs and central venous pressure. This activates the baroreflex, leading to a reduction in sympathetic nervous activity and vascular resistance. Simultaneously, metabolite accumulation may stimulate nitric oxide release, promoting vasodilation and thereby producing a significant antihypertensive effect. Compared to dynamic resistance training or aerobic exercise, this static, large-muscle-group-involved modality appears to exhibit unique advantages in blood pressure regulation. Its blood pressure-lowering effect may not only derive from the acute response during exercise but also lead to a sustained post-exercise hypotension state. Therefore, for individuals with hypertension or prehypertension, isometric training offers an efficient, low-risk, and easily monitored non-pharmacological intervention option (56).

Regarding training frequency, a regimen of three sessions per week was associated with the largest average reduction in our analysis, Carlson et al. (14) reported significantly greater systolic blood pressure reductions with thrice-weekly wall squat training compared to once-weekly sessions, without overtraining signs, supporting ACSM (57) recommendation of isometric exercise frequencies of 3–5 sessions weekly. Excessive training (>5 sessions/week) risks overtraining, causing fatigue and blunted blood pressure benefits due to inadequate recovery. Three sessions/week balance stimulus and rest, maximizing cardiovascular adaptations while ensuring long-term adherence and safety (57, 58).

Regarding intervention duration, subgroup analysis indicated that isometric training programs lasting more than 8 weeks were associated with a greater reduction in systolic blood pressure compared to those lasting 8 weeks or less. The more pronounced effect observed with longer interventions may be attributed to the gradual development of physiological adaptations, such as sustained improvements in vascular endothelial function and enhanced regulation of autonomic nervous activity, both of which contribute to systemic blood pressure lowering. These findings underscore the importance of adequate intervention duration in achieving meaningful and sustained reductions in systolic blood pressure, supporting the recommendation of isometric exercise as a long-term component of hypertension management.

Our research findings, which highlight the preferable exercise intensity at 85% HR peak, are corroborated by existing studies. These studies demonstrate that exercising at this intensity offers a sufficient physiological stimulus to the body while simultaneously circumventing undue cardiovascular stress (59, 60). When compared to prolonged high-intensity training regimens, an exercise intensity of 85% HR peak appears to mitigate the risks of both musculoskeletal and cardiovascular injuries, all the while effectively contributing to blood pressure reduction. Engaging in physical activity at 85% HR peak provides an adequate physiological boost necessary for lowering blood pressure yet steers clear of placing excessive strain on the cardiovascular system and musculoskeletal framework. In contrast, higher exercise intensities not only elevate the likelihood of injury but may also diminish individuals’ long-term commitment to regular physical activity (61, 62).

### 4.3 Diastolic blood pressure

The present meta-analysis also found that isometric exercise training significantly reduced resting diastolic blood pressure compared to control groups. Similar to the systolic pressure findings, participant characteristics and intervention parameters appeared to influence the magnitude of diastolic blood pressure reduction in subgroup analyses.

Regarding sex differences, our subgroup analysis indicated a larger average reduction in males, consistent with prior research such as Taylor et al. (51). Concerning health status, hypertensive patients experienced significantly greater reductions in diastolic blood pressure compared to normotensive individuals, suggesting enhanced responsiveness in this population (45).

Higher pre-intervention blood pressure levels also appeared to be associated with greater reductions following training. Ogbutor et al. (49) observed that individuals with baseline diastolic blood pressure ≥90 mmHg showed more pronounced improvements after isometric handgrip training, a trend consistent with our subgroup findings (63, 64). Furthermore, exercise modality may influence outcomes. Wiles et al. (46) reported that isometric wall squat training elicited greater reductions in diastolic blood pressure than other forms, likely due to the considerable cardiovascular load imposed by large-muscle static contraction, which promotes adaptations in endothelial function, arterial compliance, and autonomic regulation (65, 66). These observations highlight the importance of baseline status and exercise selection in designing targeted interventions.

Regarding intervention duration, subgroup analyses indicated that a duration exceeding 8 weeks were associated with greater reductions in diastolic blood pressure. Specifically, longer intervention length was linked to stronger effects, as programs extending beyond 8 weeks yielded a larger diastolic blood pressure reduction than those limited to 8 weeks or less—a trend consistent with the findings of Pinto et al. (33), who reported greater reductions after 12-week isometric handgrip training compared to shorter programs. The enhanced efficacy of prolonged training likely stems from cumulative adaptations in the peripheral vasculature, including gradual amelioration of endothelial function and reduced arterial stiffness, which collectively lower diastolic pressure by diminishing systemic vascular resistance. Together, these parameters—adequate frequency to provide effective stimulus without overtraining, sufficient duration to ensure sustained physiological adaptations, and higher intensity to drive cardiovascular responses—synergistically support improved blood pressure management, underscoring the utility of isometric exercise as a sustained, multi-component intervention within a comprehensive blood-pressure-lowering regimen.

Unlike the systolic blood pressure response, subgroup analyses indicated that an intensity of 95% HR peak was associated with larger reductions in diastolic blood pressure. This differential response may reflect distinct physiological regulation mechanisms between systolic and diastolic blood pressure (66). Systolic blood pressure, largely determined by cardiac contractility, stroke volume, and arterial elasticity during systole, appeared to respond more favorably to moderate intensity (85% HR peak), may improve vascular function without excessive hemodynamic stress. Conversely, diastolic blood pressure, influenced mainly by vascular tone and peripheral resistance during diastole, may benefit more markedly from higher intensity (95% HR peak) (61). The stronger mechanical and metabolic stimulus at higher intensity could promote greater nitric oxide release, enhance endothelium-dependent vasodilation, and improve vascular compliance, thereby reducing peripheral resistance more effectively during diastole (67). Additionally, higher-intensity exercise may induce more pronounced adaptations in vascular smooth muscle sensitivity and autonomic balance, further supporting diastolic blood pressure lowering (68, 69).

In conclusion, isometric exercise training for diastolic blood pressure reduction is influenced by multiple factors, with 95% HR peak exercise intensity demonstrating unique advantages. These findings provide important theoretical basis for developing targeted isometric exercise protocols specifically aimed at diastolic blood pressure control.

### 4.4 Limitations

This research presents several significant constraints. First, substantial heterogeneity was observed across studies, which may stem from variations in blood pressure measurement methods (e.g., office, home, or ambulatory monitoring), differing degrees of intervention supervision (supervised vs. home-based), and variability in participant adherence and compliance. These factors could contribute to the variability in individual study outcomes and affect the precision of the pooled estimates.

Second, while our study encompassed relatively broad participant demographics including young healthy adults, middle-aged and older individuals, women, and prehypertensive populations, certain special populations might have been underrepresented. For instance, patients with severe cardiovascular diseases, renal disorders, or other chronic conditions with unstable clinical status were likely excluded from original trials due to elevated exercise risks. Consequently, the applicability of our findings to these special populations requires further validation.

Furthermore, most included studies primarily focused on short-term effects, with relatively scarce long-term follow-up data. Given that blood pressure management constitutes a long-term process, the sustained control efficacy and potential long-term adverse effects of maintained isometric exercise training remain unclear. Therefore, our study cannot comprehensively evaluate the effectiveness and safety of isometric exercise training for long-term blood pressure management.

Additionally, although we allowed the inclusion of participants on stable antihypertensive medication provided it was balanced between groups, variability in medication types and dosages across studies may still represent a potential confounding factor. Future studies should strive for more detailed reporting and control of pharmacological backgrounds to further clarify the independent effect of isometric exercise on blood pressure.

## 5 Conclusion

Isometric exercise training was associated with significant improvements in resting blood pressure. Subgroup analyses suggested that the greatest reductions appeared to be more pronounced in males and hypertensive patients, and were associated with the wall squat modality. A regimen of three sessions per week for over 8 weeks was linked to larger effect sizes in our analysis. Regarding exercise intensity, while higher intensities were associated with improvements, particularly for diastolic blood pressure, the preferable stimulus may differ between systolic and diastolic components. Therefore, the intensity should be tailored, with consideration that moderate to high intensities (in the range of 85–95% HR peak) appear promising for inducing clinically relevant blood pressure-lowering effects. Such training may serve as a beneficial adjunct exercise regimen.

## Data Availability

The original contributions presented in the study are included in the article/Supplementary material, further inquiries can be directed to the corresponding authors.
