# Correction: Effects of isometric training based on the entire population on blood pressure regulation: systematic review and meta-analysis of randomized controlled trials

**DOI:** 10.3389/fpubh.2026.1866759

**Published:** 2026-05-26

**Authors:** Yu Yan, Chu Sun, Lidan Pan, Hui Ma, Huisong Xie

**Affiliations:** 1 College of Education, Beijing Sport University, Beijing, China; 2 The School of International Education and Exchange, Beijing Sport University, Beijing, China; 3 Sports Coaching College, Beijing Sport University, Beijing, China

**Keywords:** cardiovascular, diastolic blood pressure, isometric exercise training, meta-analysis, systolic blood pressure

There was a mistake in Figures 2–5, 7, 8 and Tables 2, 3, 5, 6 as published. A data entry error was made when extracting results from “Danielsen MB, Andersen S, Ryg J, Bruun NH, Madeleine P, Jorgensen MG. Effect of a home-based isometric handgrip training programme on systolic blood pressure in adults: a randomised assessor-blinded trial. J Sports Sci (2023) 41:1815–1823”. The blood pressure was incorrectly recorded as “Experimental (mean ± SD): −13 ± 14.626, *n* = 24, Control (mean ± SD): −13.8 ± 13,744 *n* = 24” instead of “Experimental (mean ± SD): −6.2 ± 13.474, *n* = 21, Control (mean ± SD): −12.7 ± 14.870 *n* = 22”. This led to minor numerical discrepancies in the pooled estimates. However, after correcting the error and re-analyzing all data (forest plot, meta-regression, funnel plot, sensitivity analysis), all effect estimates and conclusions remained unchanged. The mistake was an inadvertent transcription error and does not affect the scientific validity of the meta-analysis. The corrected Figures 2–5, 7, 8 and Tables 2, 3, 5, 6 appear below.

**Figure 2:**
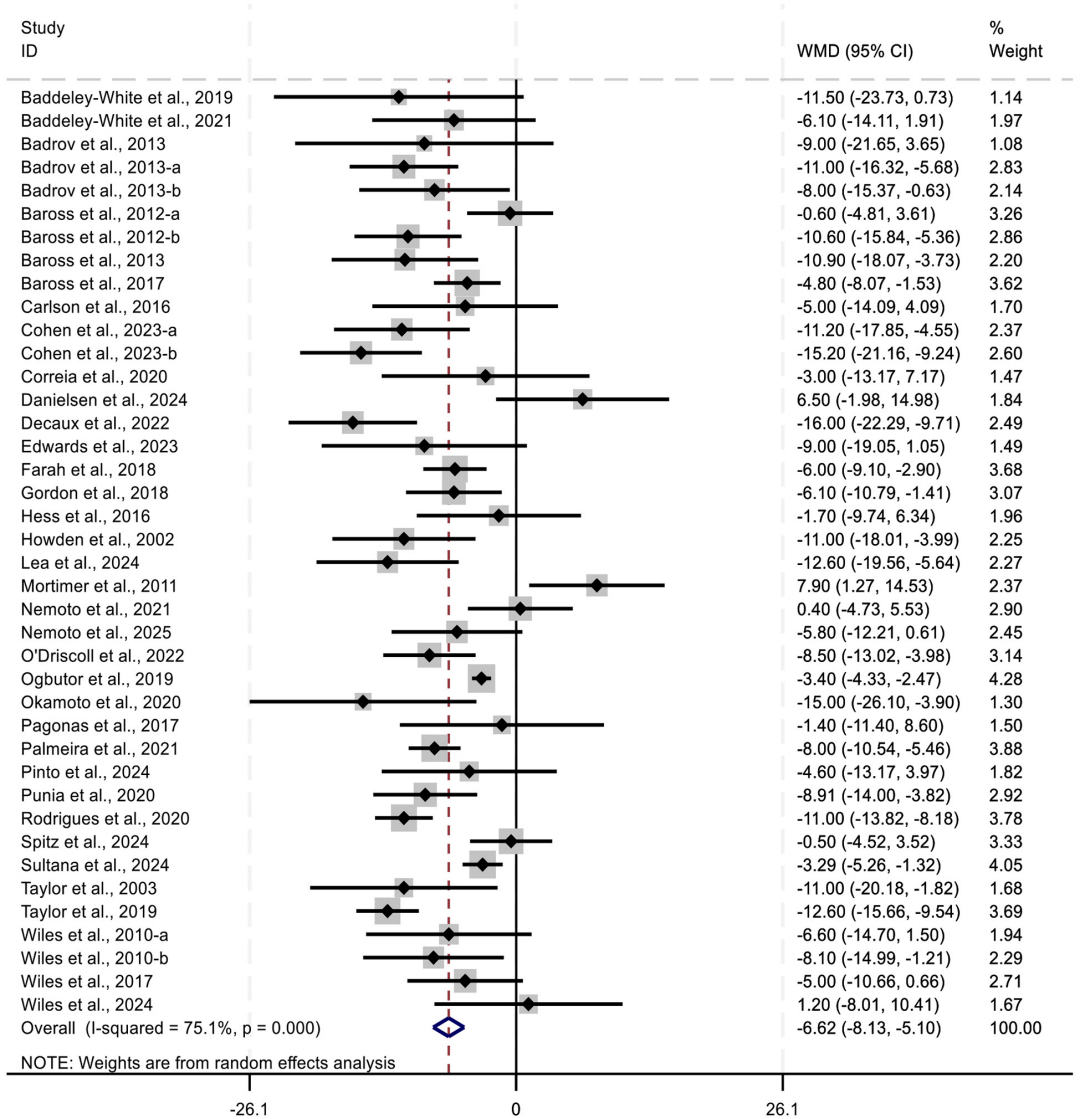
A meta-analysis of the effects of isometric training on systolic blood pressure.

**Figure 3:**
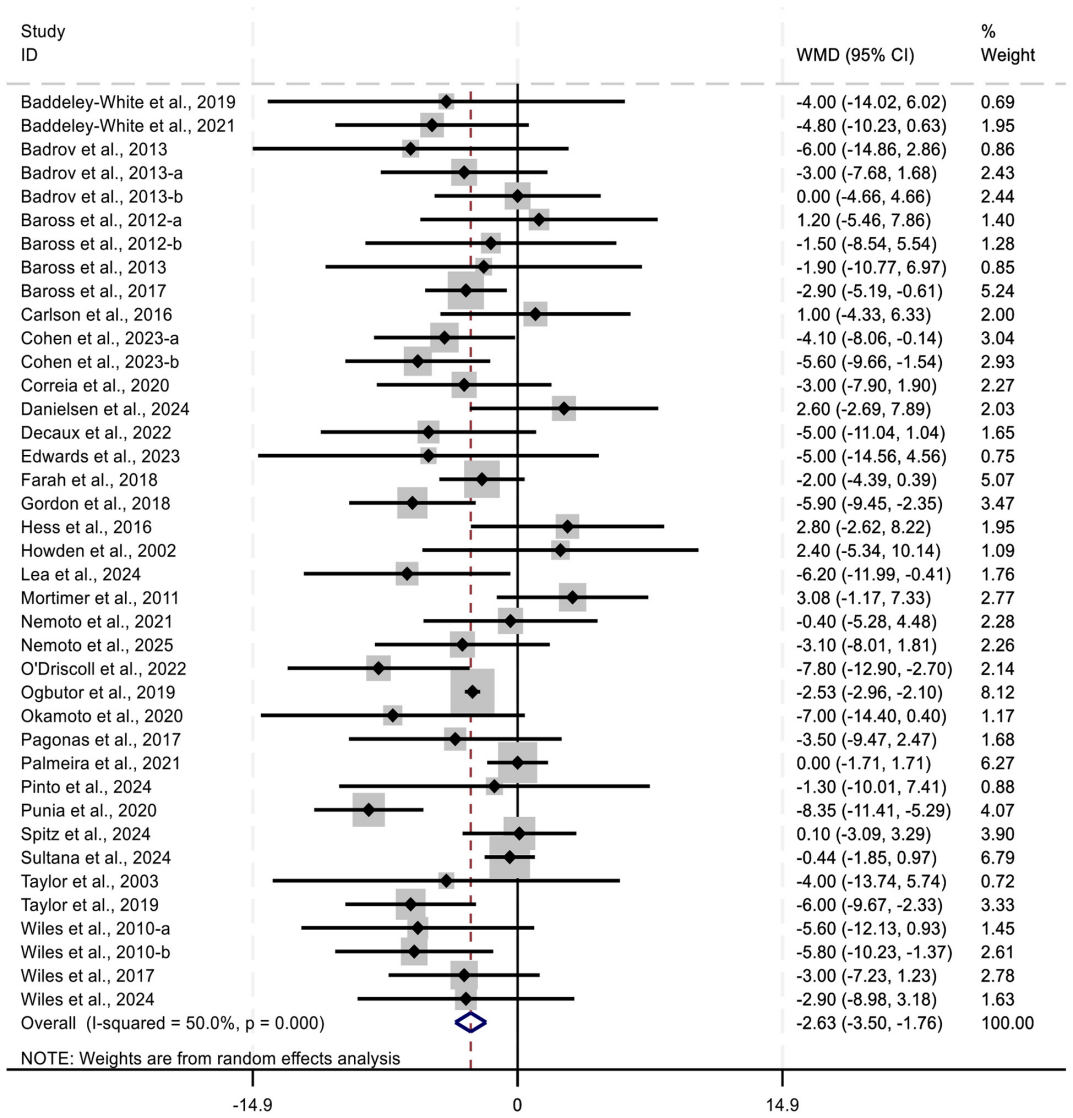
A meta-analysis of the effects of isometric training on diastolic blood pressure.

**Figure 4:**
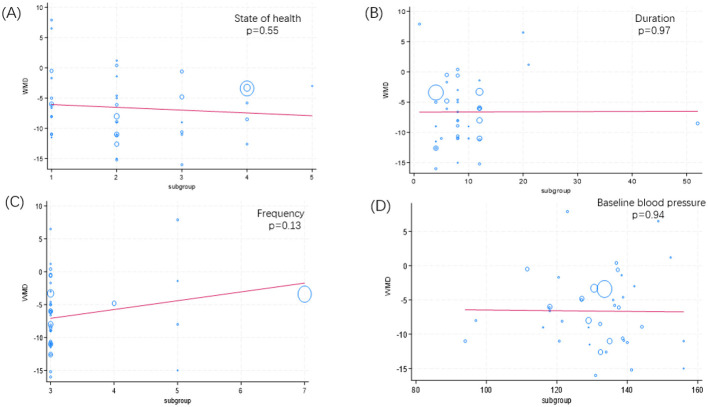
Meta-regression analysis results for isometric exercise training on SBP. **(A)** State of health; **(B)** Duration; **(C)** Frequency; **(D)** Baseline blood pressure.

**Figure 5:**
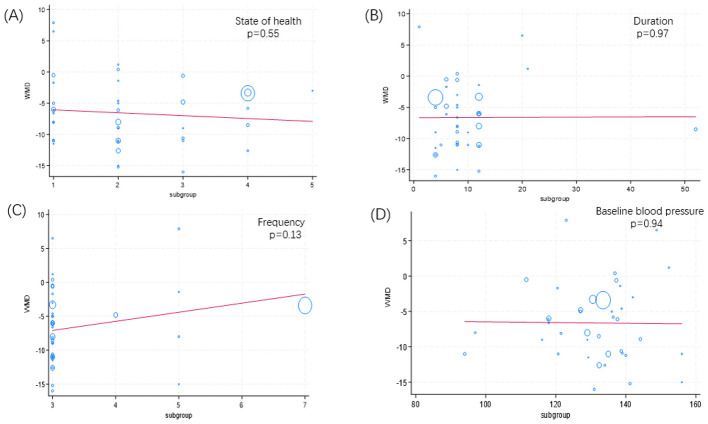
Meta-regression analysis results for isometric exercise training on DBP. **(A)** State of health; **(B)** Duration; **(C)** Frequency; **(D)** Baseline blood pressure.

**Figure 7:**
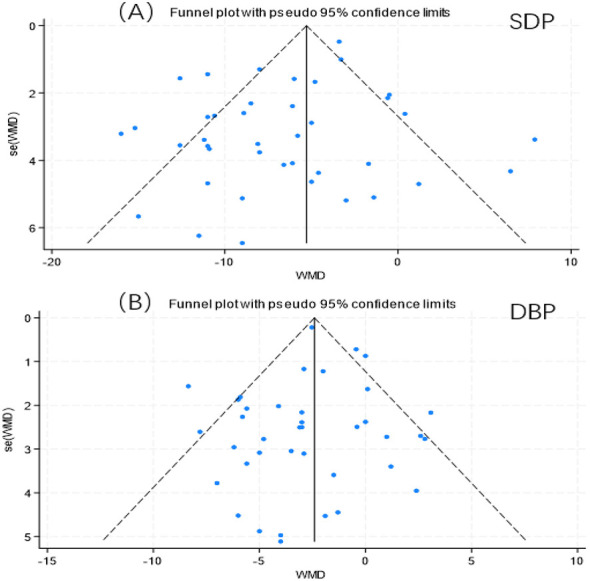
Results of funnel plot. **(A)** Results of funnel plot on SBP; **(B)** Results of funnel plot on DBP.

**Figure 8:**
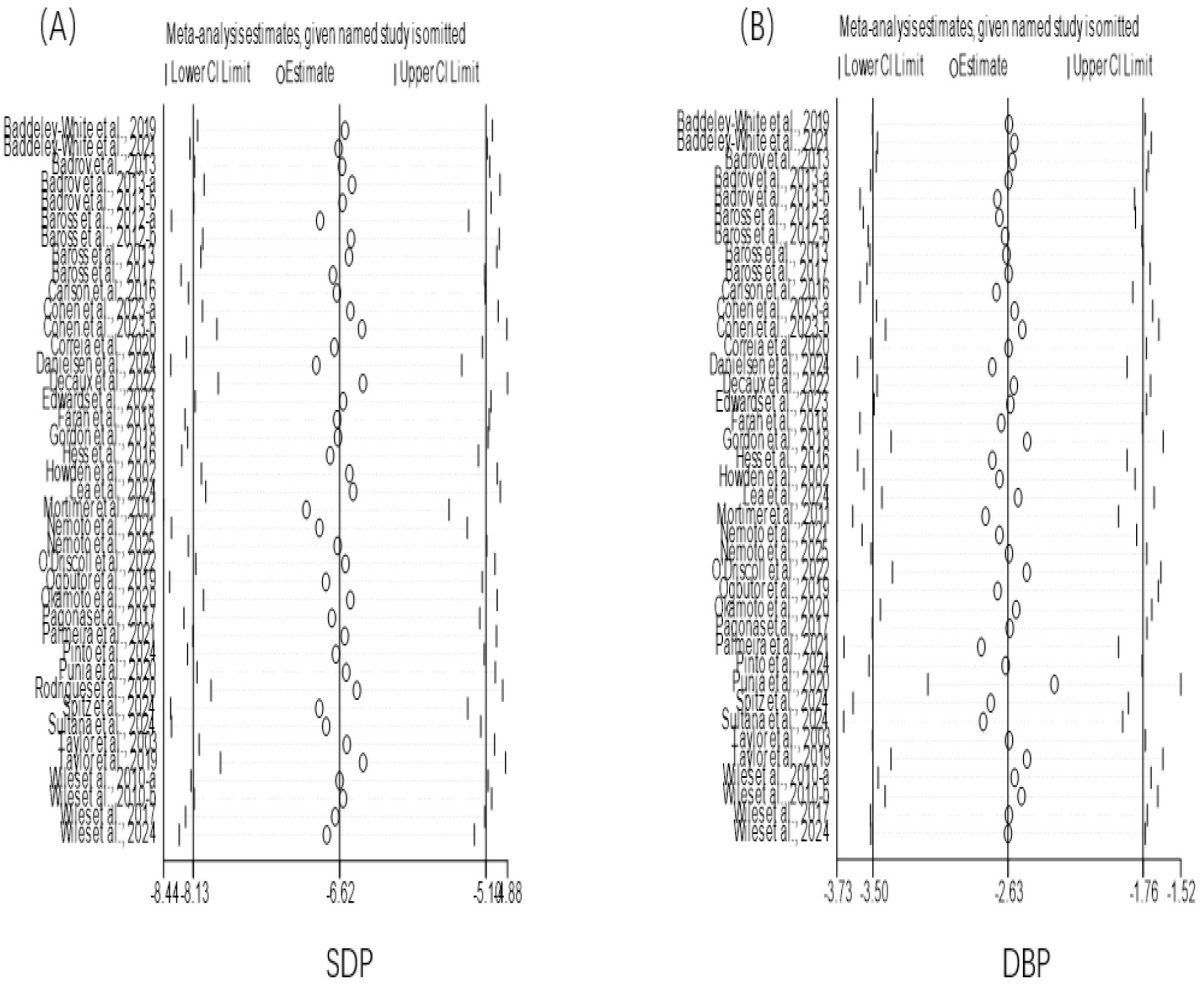
Results of sensitivity analyses. **(A)** Results of sensitivity analyses on SBP; **(B)** Results of sensitivity analyses on SBP.

**Table 2:** Subgroup analysis results for systolic blood pressure (random-effects model).

Subgroup	*K* (*n*)	MD (95% CI) mmHg	*p*_d_	*p*_m_	*I* ^2^
**Gender**				*p* < 0.0001	71%
Male	9 (123)	−6.23 [−8.81, −3.66]	*p* < 0.00001		55%
Female	3 (32)	−5.91 [−8.76, −3.06]	*p* = 0.53		90%
**Health status**				*p* < 0.00001	76%
Healthy adults	13 (183)	−4.57 [−7.66, −1.48]	*p* = 0.0002		68%
Sedentary	6 (68)	−8.22 [−12.80, −3.63]	*p* = 0.0004		77%
Pre-hypertensive	5 (281)	−5.14 [−7.41, −2.87]	*p* < 0.00001		65%
Hypertensive	15 (278)	−8.22 [−10.57, −5.86]	*p* < 0.00001		62%
**Baseline blood pressure**				*p* < 0.00001	75%
< 120	7 (117)	−6.16 [−9.14, −3.18]	*p* < 0.00001		71%
≥120–140	25 (561)	−6.45 [−8.30, −4.60]	*p* < 0.00001		79%
≥140	8 (161)	−7.32 [−12.46, −2.18]	*p* = 0.0009		60%
**Form of exercise**				*p* < 0.00001	76%
BILT	6 (61)	−7.63 [−11.74, −3.52]	*p* = 0.0003		62%
IWST	8 (125)	−10.29 [−13.60, −6.98]	*p* < 0.00001		61%
IHG	25 (643)	−5.24 [−6.93, −3.55]	*p* < 0.00001		72%
**Frequency (per week)**				*p* < 0.00001	71%
3	34 (572)	−7.19 [−8.85, −5.53]	*p* < 0.00001		69%
5	4 (55)	−3.66 [−13.61, 6.29]	*p* = 0.47		82%
**Duration**				*p* < 0.00001	75%
≤ 8	26 (582)	−6.55 [−8.59, −4.51]	*p* < 0.00001		76%
>8	14 (257)	−6.82 [−9.20, −4.44]	*p* < 0.0001		72%
**Intensity**				*p* < 0.00001	77%
20%MVC	2 (21)	−7.09 [−12.96, −1.23]	*p* = 0.02		59%
30%MVC	24 (630)	−6.26 [−8.27, −4.26]	*p* < 0.00001		81%
70%HR peak	2 (21)	−2.56 [−8.08, 2.95]	*p* = 0.36		40%
85%HR peak	3 (31)	−9.99 [−13.60, −6.38]	*p* < 0.00001		0%
95%HR peak	6 (74)	−8.69 [−13.02, −4.37]	*p* < 0.0001		60%

*K* (*n*), the number of studies included in the pooled effect analysis (total number of participants across pooled studies); *p*_d_, the *p*-value used to assess differences in effect size between subgroups; *p*_m_, the *p*-value for the heterogeneity test.

**Table 3:** Subgroup analysis results for diastolic blood pressure (random-effects model).

Subgroup	*K* (*n*)	MD (95% CI) mmHg	*p_*d*_*	*p_*m*_*	*I^2^*
**Gender**				*p* = 0.02	45%
Male	9 (123)	−3.07 [−5.33, −0.82]	*p* = 0.008		46%
Female	3 (32)	0.14 [−3.35, 3.63]	*p* = 0.94		44%
**Health status**				*p* = 0.002	51%
Healthy adults	13 (183)	−1.40 [−3.01, 0.21]	*p* = 0.11		35%
Sedentary	6 (68)	−2.45 [−4.32, −0.59]	*p* = 0.01		0%
Pre-hypertensive	5 (281)	−2.74 [−4.60, −0.88]	*p* = 0.004		71%
Hypertensive	14 (262)	−3.87 [−5.88, −1.87]	*p* = 0.0002		64%
**Baseline blood pressure**				*p* = 0.0002	50%
< 80	19 (285)	−1.51 [−2.75, −0.28]	*p* = 0.02		12%
≥80–89	16 (482)	2.60 [−2.69, 7.89]	*p* =0.001		59%
≥90	4 (56)	−5.22 [−8.64, −1.79]	*p* = 0.003		65%
**Form of exercise**				*p* = 0.0002	51%
BILT	6 (61)	−2.65 [−5.50, 0.21]	*p* = 0.07		13%
IWST	8 (125)	−5.26 [−7.02, −3.51]	*p* < 0.00001		0%
IHG	24 (627)	−1.96 [−3.00, −0.93]	*p* = 0.0002		59%
**Frequency (per week)**				*p* = 0.0002	51%
3	33 (572)	−2.89 [−4.02, −1.76]	*p* = 0.0004		52%
5	5 (67)	−1.58 [−4.54, 1.38]	*p* = 0.30		54%
**Duration**				*p* = 0.0002	50%
≤8	26 (583)	−2.67 [−3.80, −1.55]	*p* < 0.00001		44%
>8	13 (240)	−2.70 [−4.25, −1.16]	*p* = 0.007		56%
**Intensity**				*p* = 0.0002	52%
20%MVC	2 (21)	−1.59 [−6.07, 2.88]	*p* = 0.49		40%
30%MVC	23 (613)	−2.45 [−3.54, −1.35]	*p* < 0.0001		63%
70%HR peak	2 (21)	−2.23 [−8.90, 4.43]	*p* = 0.51		51%
85%HR peak	3 (31)	−4.17 [−7.63, −0.72]	*p* = 0.02		0%
95%HR peak	6 (74)	−4.87 [−7.17, −2.57]	*p* < 0.0001		0%

*K* (*n*), the number of studies included in the pooled effect analysis (total number of participants across pooled studies); *p*_d_, the *p*-value used to assess differences in effect size between subgroupps; *p*_m_, the *p*-value for the heterogeneity test.

**Table 5:** Results of Egger's test (SBP).

Std_Eff	Coefficient	Std. Err.	*t*	*P* > |*t*|	[95% conf. interval]
Slope	−3.796443	0.9294134	−4.08	0.000	(−5.677942, −1.914944)
Bias	−0.9941771	0.4792466	−2.07	0.045	(−1.964361, −0.023993)

Std. Err, standard error; *t, t*-test statistic; *p*, probability.

**Table 6:** Results of Egger's test (DBP).

Std_Eff	Coefficient	Std. err.	*t*	*P* > |*t*|	[95% conf. interval]
Slope	−2.301857	0.3278464	−7.02	0.000	(−2.966137, −1.637577)
Bias	−0.1513385	0.2905962	−0.52	0.606	(−0.7401422, 0.4374653)

Std. Err, standard error; *t, t*-test statistic; *p*, probability.

In the abstract, “(WMD, −6.72; 95% CI, −8.21 to −5.23, *p* < 0.0001, *I*^2^ = 74%) and DBP (WMD, −2.72; 95% CI, −3.57 to −1.87, *p* < 0.0001, *I*^2^ = 48%)”. This has been corrected to read:

“(WMD, −6.62; 95% CI, −8.13 to −5.10, *p* < 0.0001, *I*^2^ = 75%) and DBP (WMD, −2.63; 95% CI, −3.50 to −1.76, *p* < 0.0001, *I*^2^ = 50%)”

“Due to the data entry error described in the Error in figure/table section above, the following numerical values in the **Results** section have been corrected.”

A correction has been made to the section **3 Results**, ***3.3 Main effect***, paragraph 1:

“Isometric training can effectively reduce systolic blood pressure (WMD, −6.62; 95% CI, −8.13 to −5.10, *p* < 0.0001, *I*^2^ = 75%, Figure 2) and diastolic blood pressure (WMD, −2.63; 95% CI, −3.50 to −1.76, *p* < 0.0001, *I*^2^ = 50%, Figure 3). Meta-analysis indicated substantial heterogeneity in both systolic and diastolic blood pressure outcomes. To explore potential sources of this variability and identify modifiable exercise-related factors, additional analyses including meta-regression, subgroup analysis, and sensitivity analysis were performed. It should be noted that the following subgroup analyses are exploratory in nature, aimed at generating hypotheses, and their findings are susceptible to ecological bias. They should not be interpreted as definitive evidence of causal relationships.”

A correction has been made to the section **3 Results**, ***3.4 Meta-regression***, paragraphs 1 and 2:

“As shown in Figure 4, the results of the meta-regression suggest no significant association between health status (*p* = 0.55, Figure 4A), duration (*p* = 0.97, Figure 4B), frequency (*p* = 0.13, Figure 4C), baseline blood pressure (*p* = 0.94, Figure 4D), and the reduction in systolic blood pressure achieved through isometric training.

As shown in Figure 5, meta-regression analysis indicates no significant association between health status (*p* = 0.40, Figure 5A), duration (*p* = 0.22, Figure 5B), frequency (*p* = 0.52, Figure 5C), baseline blood pressure (*p* = 0.12, Figure 5D), and the reduction in diastolic blood pressure achieved through isometric training.”

A correction has been made to the section **3 Results** ***3.5 Subgroup analysis*** *3.5.1 Systolic blood pressure*, paragraph 3:

“Furthermore, the greatest blood pressure reduction was associated with isometric exercise regimens that involved wall squat training (WMD, −10.29; 95% CI, −13.60 to −6.98, *p* < 0.00001, *I*^2^ = 61%), three sessions weekly (WMD: −7.19; 95% CI, −8.85 to −5.53, *p* < 0.00001, *I*^2^ = 69%), an intensity of 85% HR peak (WMD, −9.99; 95% CI, −13.60 to −6.38, *p* < 0.00001, *I*^2^ = 0%), and a duration of more than 8 weeks (WMD, −6.82; 95% CI, −9.20 to −4.44, *p* < 0.0001, *I*^2^ = 72%).”

A correction has been made to the section **3 Results** ***3.5 Subgroup analysis*** *3.5.2 Diastolic blood pressure*, paragraph 3:

“Furthermore, the greatest blood pressure-lowering outcomes were associated with the following exercise regimens: wall squat training (WMD, −5.26; 95% CI, −7.02 to −3.51, *p* < 0.00001, *I*^2^ = 0%), three sessions weekly (WMD, −2.89; 95% CI, −4.02 to −1.76, *p* = 0.0004, *I*^2^ = 52%), an intensity of 95% HR peak (WMD, −4.87; 95% CI, −7.17 to −2.57), *p* < 0.0001, *I*^2^ = 0%), and an intervention duration exceeding 8 weeks (WMD, −2.70; 95% CI, −4.25 to −1.16, *p* = 0.007, *I*^2^ = 56%).”

A correction has been made to the section **3 Results** ***3.8 Publication bias***, paragraphs 1, 2 and 3:

“Funnel plots were employed to analyses publication bias. Visual inspection of the funnel plot (Figure 7A) combined with the Egger test (*t* = −2.07, *p* = 0.045, Table 5). This suggests the potential presence of publication bias or small-study effects, where smaller studies showing larger effect sizes might be more likely to be published.

To estimate and adjust for the potential impact of any missing studies, we applied the Trim and Fill method. The raw pooled effect size for isometric exercise on systolic blood pressure was −6.62 mmHg, and it remained unchanged after adjustment (−6.62 mmHg, 95% CI: −8.131 to −5.099, *p* < 0.0001). This analysis indicated that no studies needed to be imputed to achieve symmetry in the funnel plot. Thus, the pooled effect estimate for systolic blood pressure reduction was robust to this adjustment. While the Egger's test indicates asymmetry, the lack of imputation by the Trim and Fill procedure suggests that any potential publication bias may not be substantial enough to qualitatively alter the main conclusion that isometric exercise significantly reduces systolic blood pressure. Nevertheless, the possibility of a modest overestimation of the true effect size due to small-study effects cannot be ruled out.

Similarly, funnel plots were employed to detect publication bias across all 39 trials for diastolic blood pressure. Visual inspection of the funnel plots (Figure 7B) and the Egger test (*t* = −0.52, *p* = 0.606, Table 6) indicated no evidence of publication bias, rendering the results suitable for meta-analysis.”

The original version of this article has been updated.

