# Supplementary figures and images for: Effects of isometric training based on the entire population on blood pressure regulation: systematic review and meta-analysis of randomized controlled trials
Source: Front Public Health. 2026 Mar 2;14:1774541. doi: 10.3389/fpubh.2026.1774541 (PMC12989405; doi:10.3389/fpubh.2026.1774541)

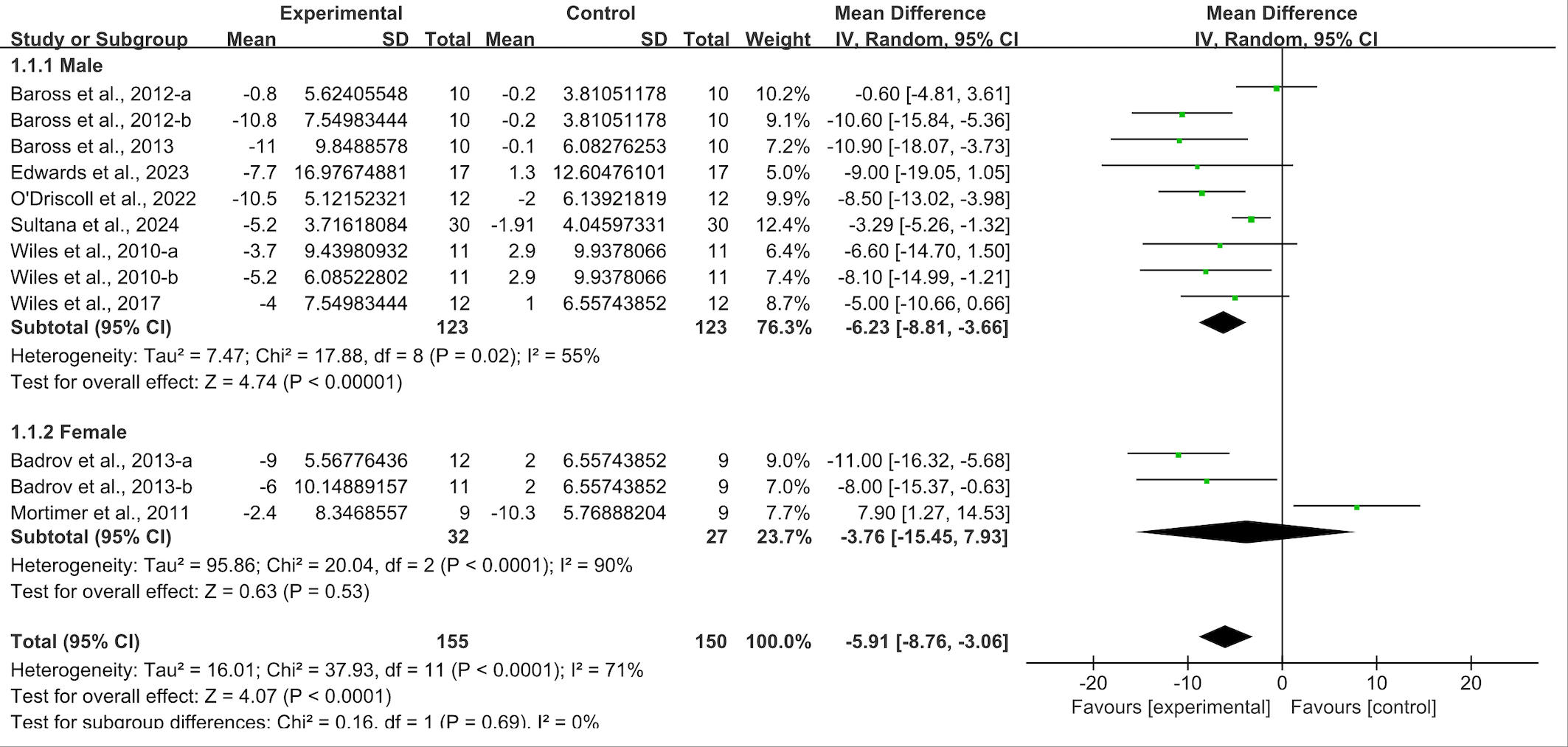

Supplement: Supplementary file 1 [file Supplementary_file_1.zip › Supplementary Materials/Figure 1.tiff]

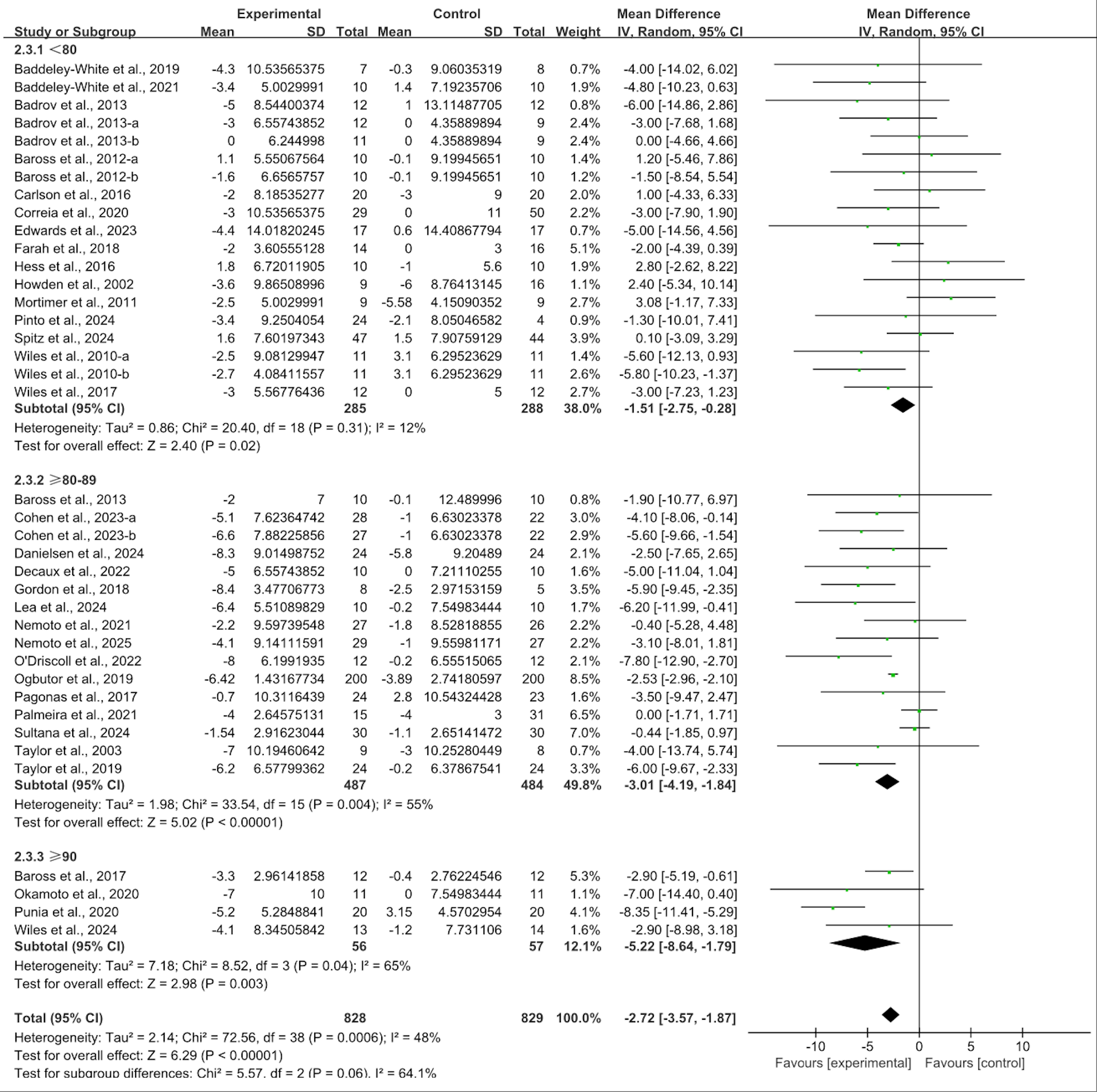

Supplement: Supplementary file 1 [file Supplementary_file_1.zip › Supplementary Materials/Figure 10.tiff]

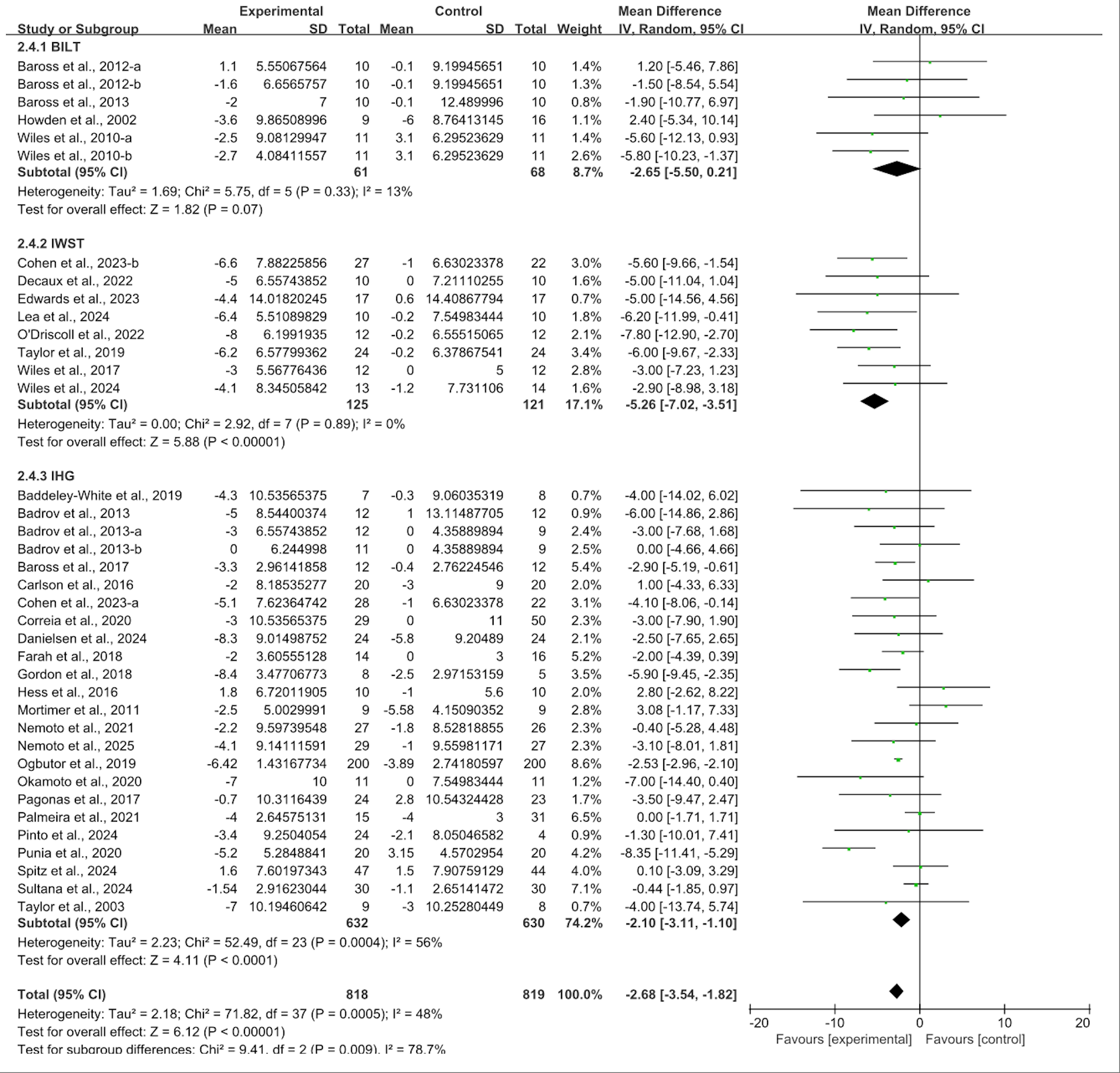

Supplement: Supplementary file 1 [file Supplementary_file_1.zip › Supplementary Materials/Figure 11.tiff]

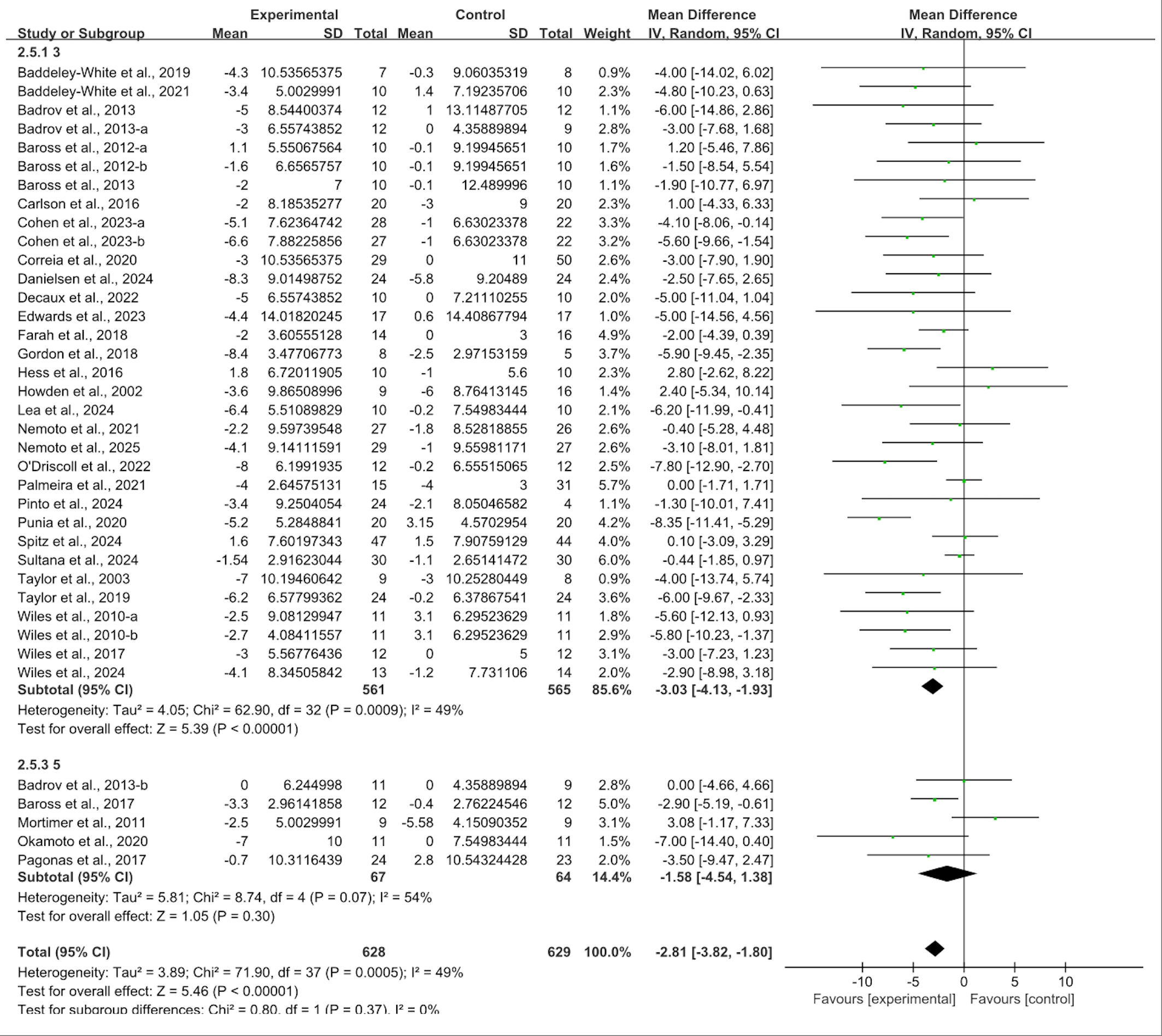

Supplement: Supplementary file 1 [file Supplementary_file_1.zip › Supplementary Materials/Figure 12.tiff]

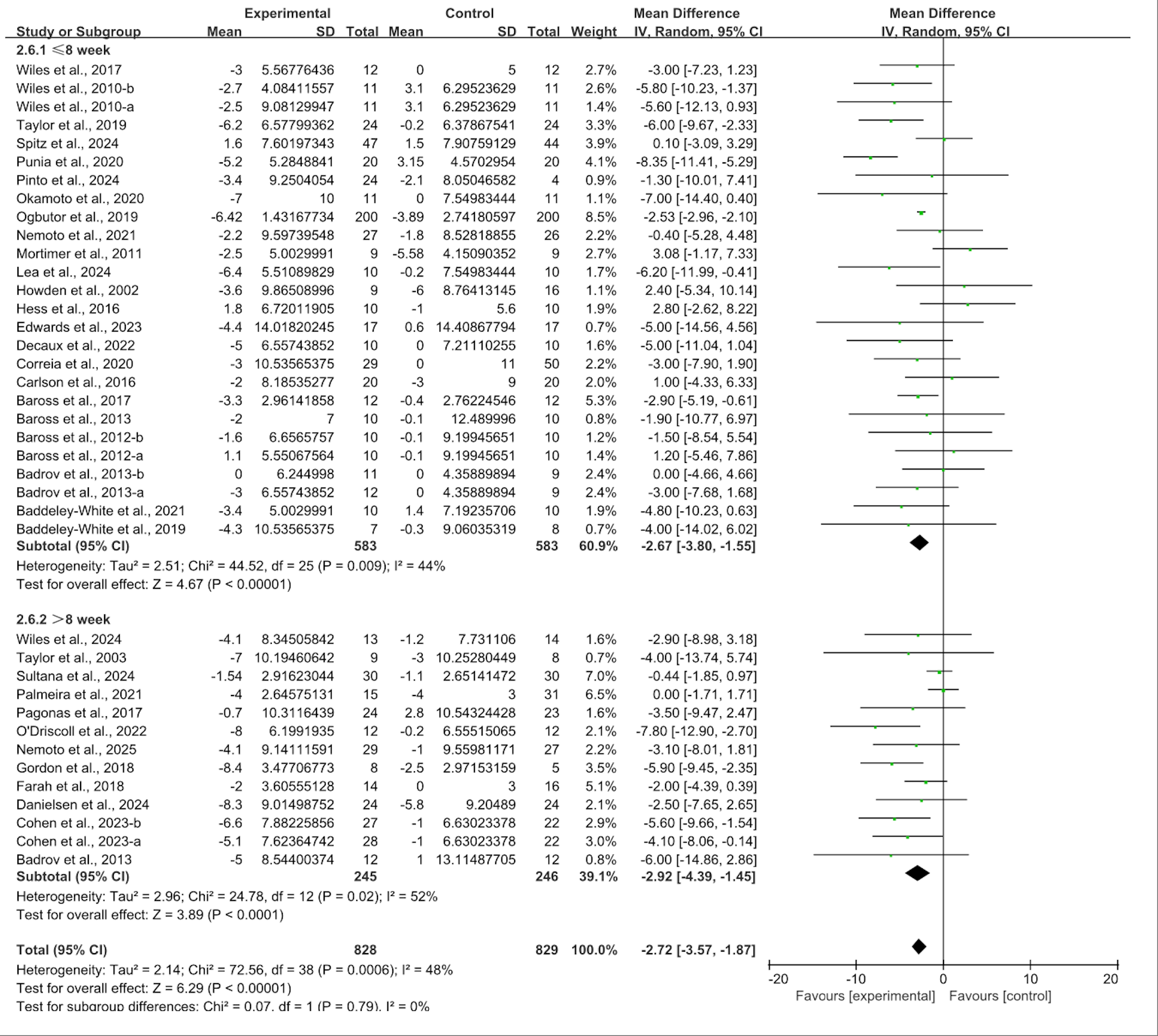

Supplement: Supplementary file 1 [file Supplementary_file_1.zip › Supplementary Materials/Figure 13.tiff]

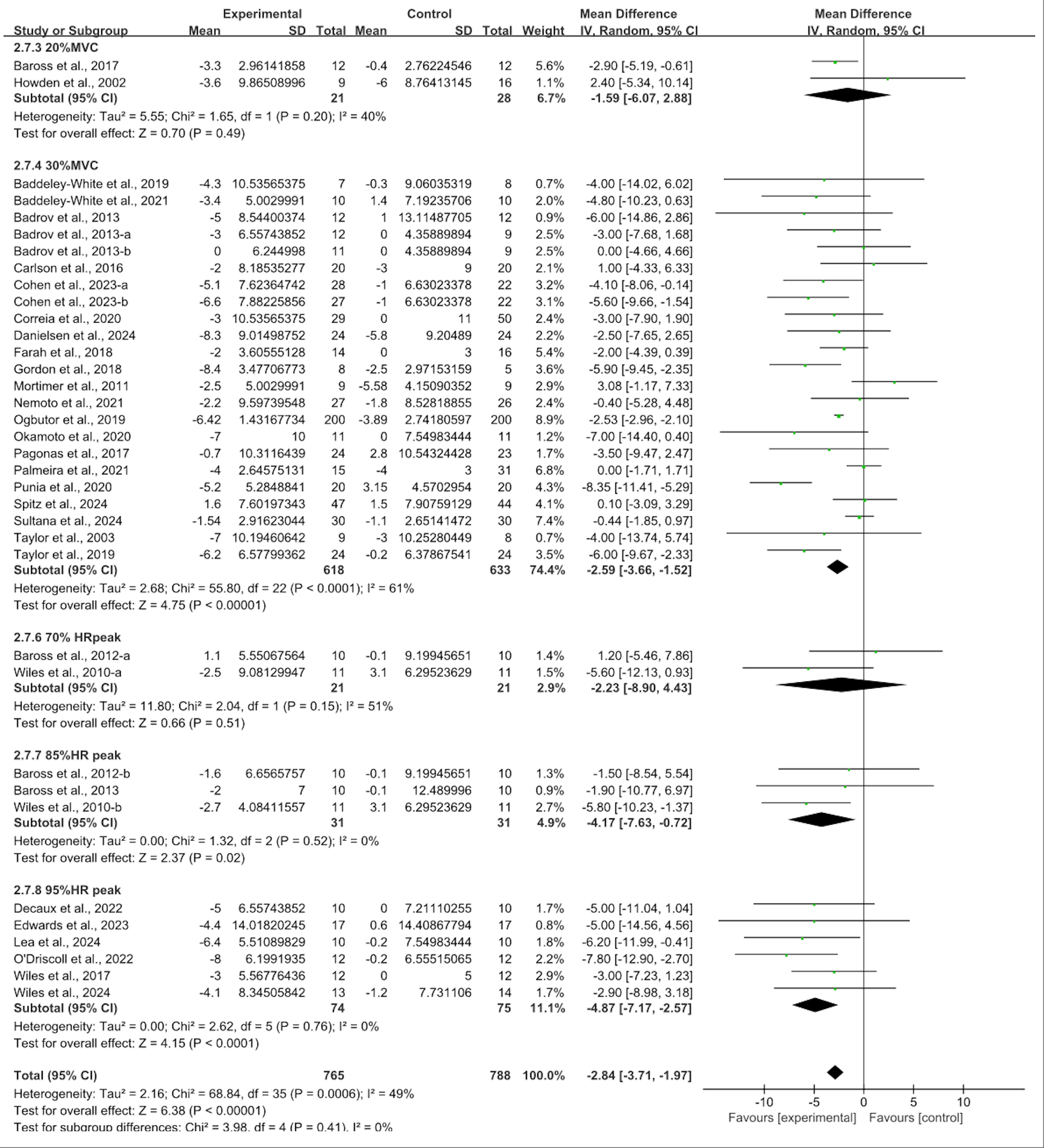

Supplement: Supplementary file 1 [file Supplementary_file_1.zip › Supplementary Materials/Figure 14.tiff]

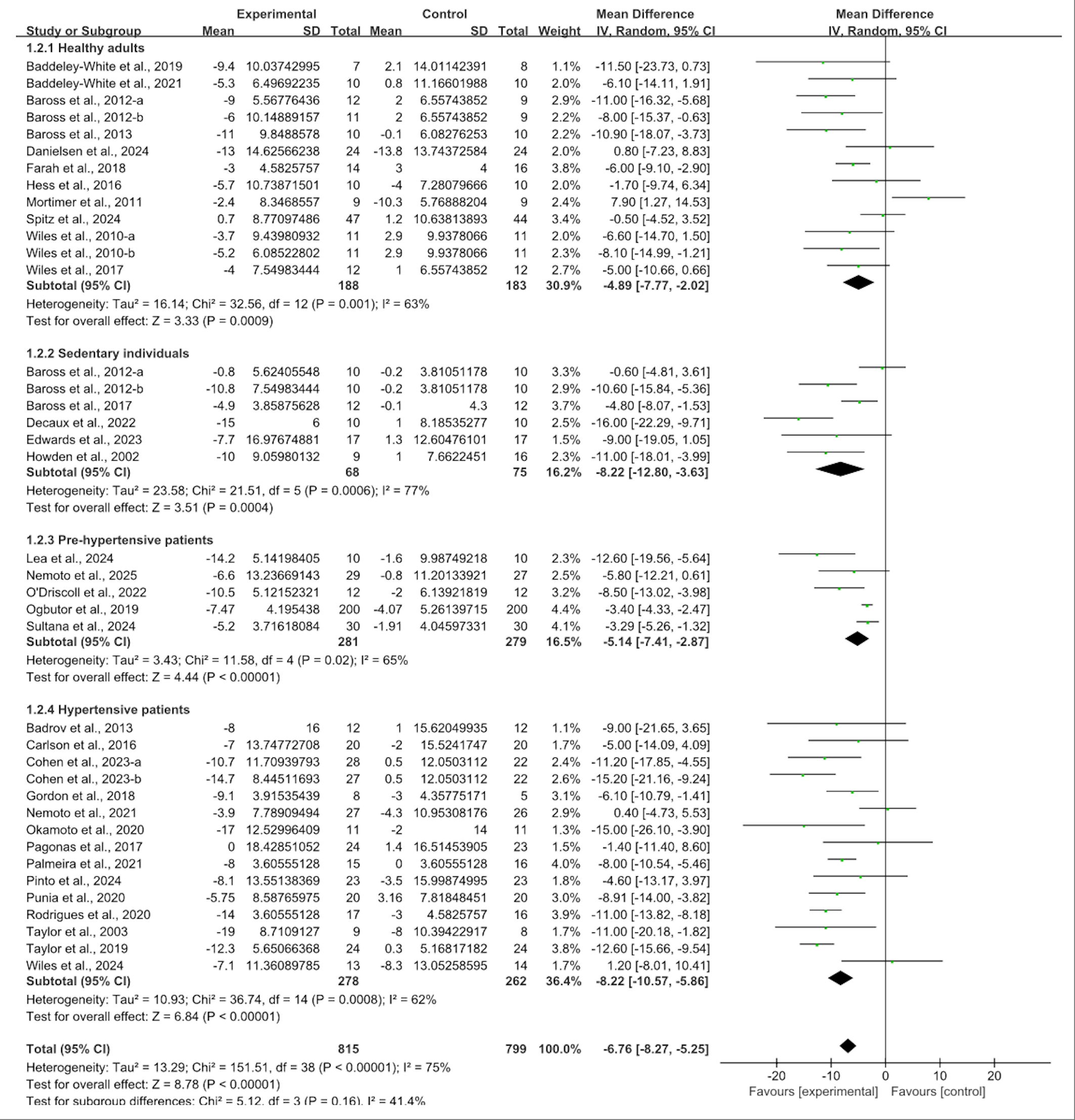

Supplement: Supplementary file 1 [file Supplementary_file_1.zip › Supplementary Materials/Figure 2.tiff]

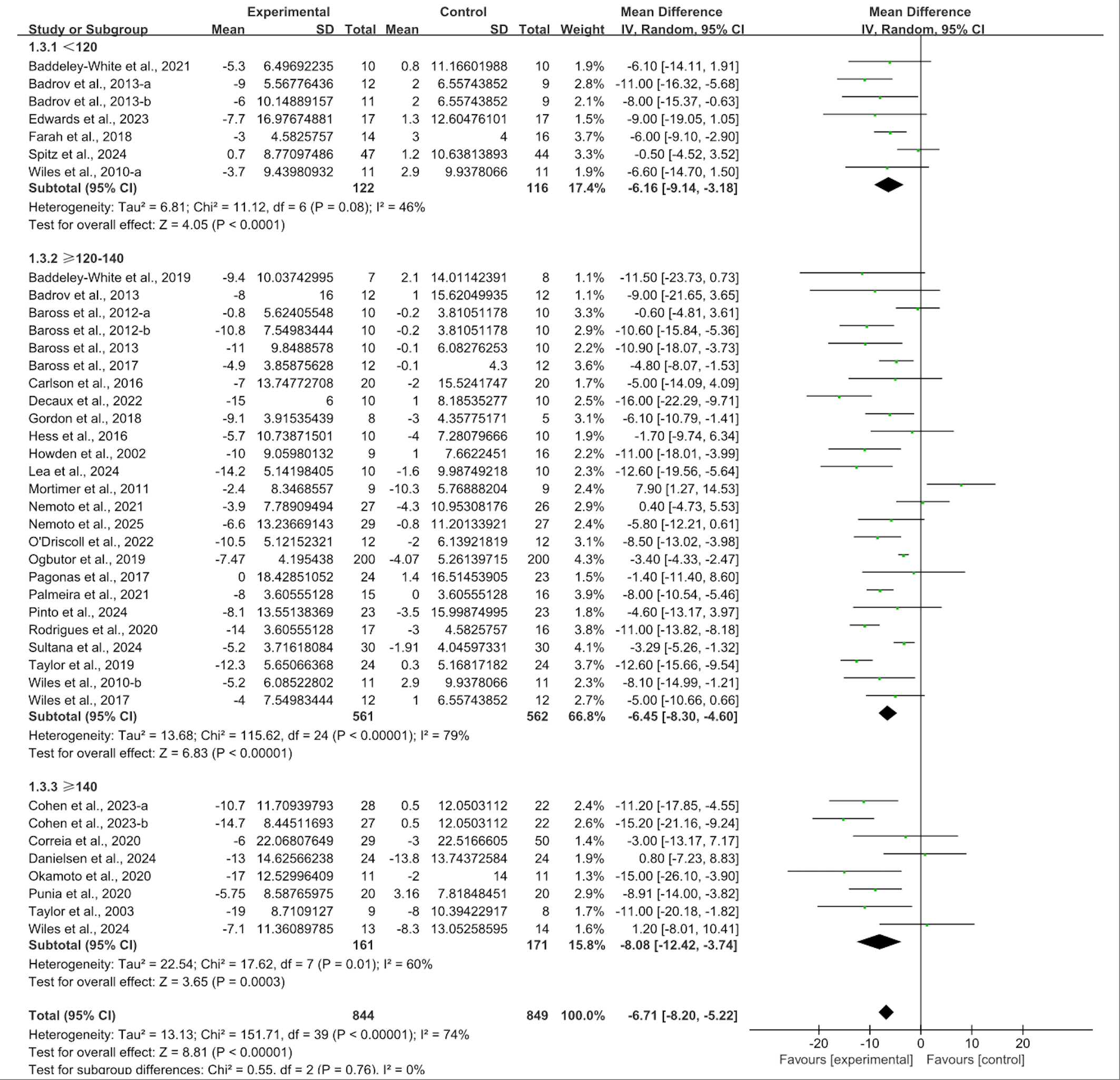

Supplement: Supplementary file 1 [file Supplementary_file_1.zip › Supplementary Materials/Figure 3.tiff]

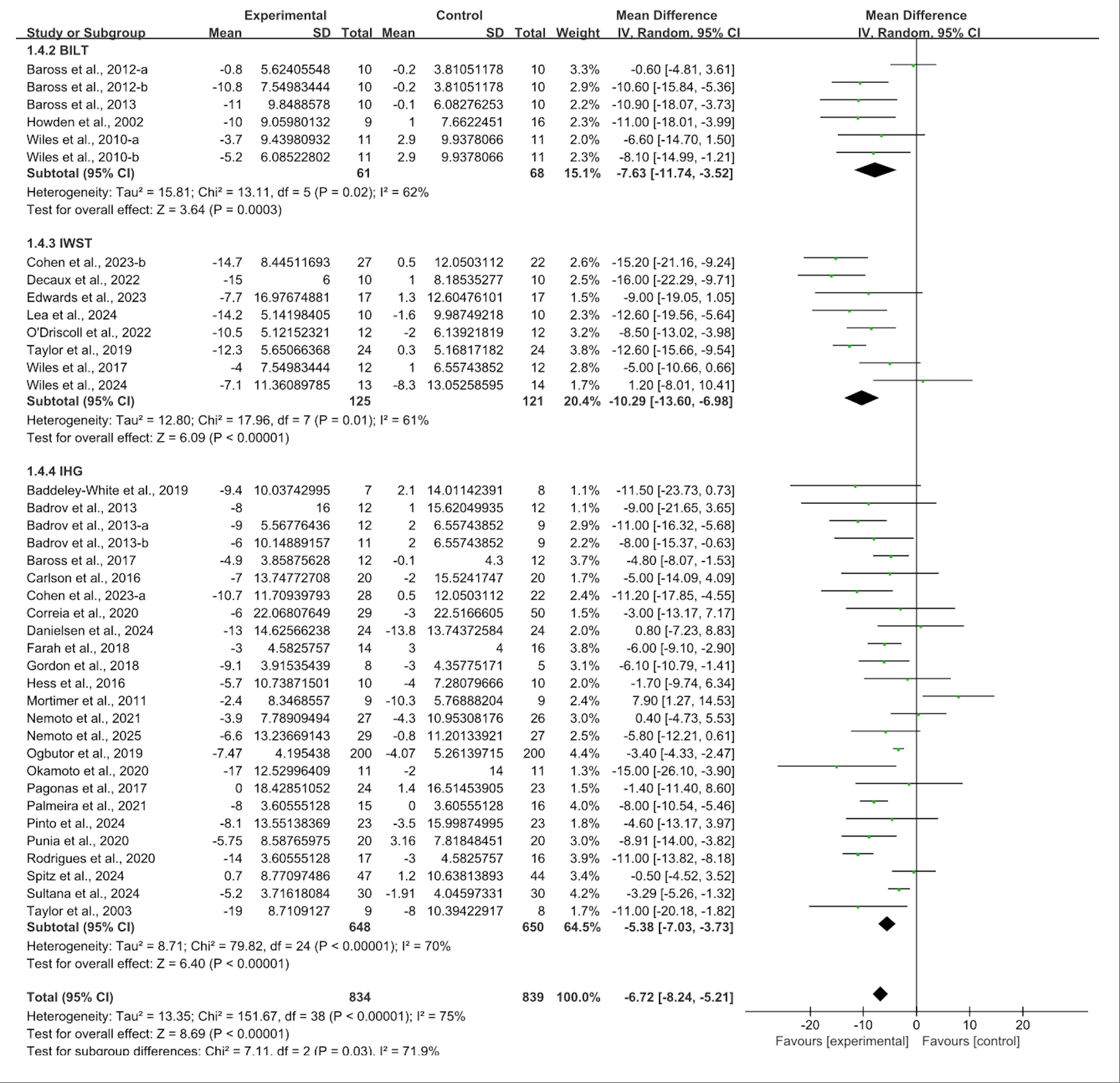

Supplement: Supplementary file 1 [file Supplementary_file_1.zip › Supplementary Materials/Figure 4.tiff]

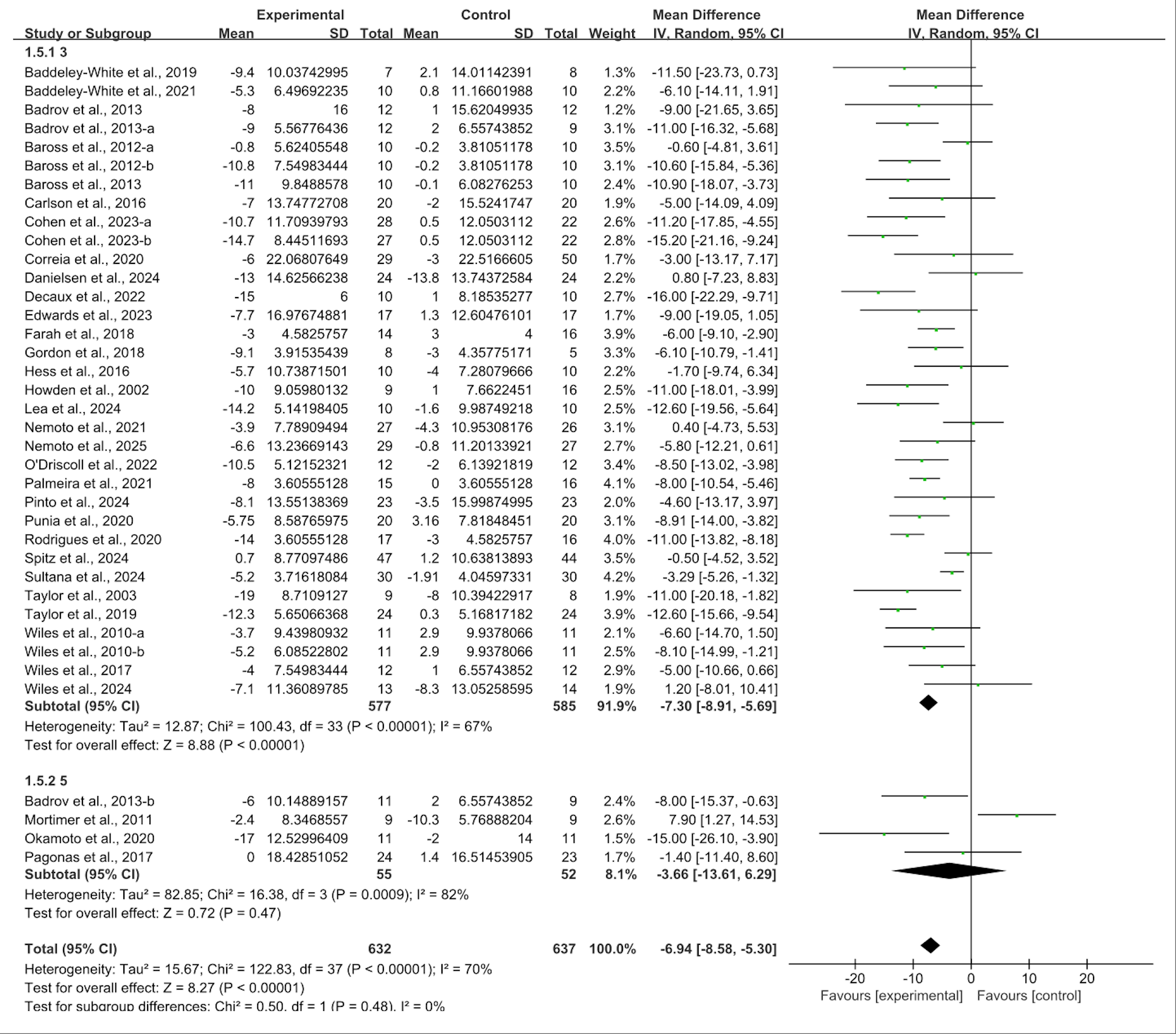

Supplement: Supplementary file 1 [file Supplementary_file_1.zip › Supplementary Materials/Figure 5.tiff]

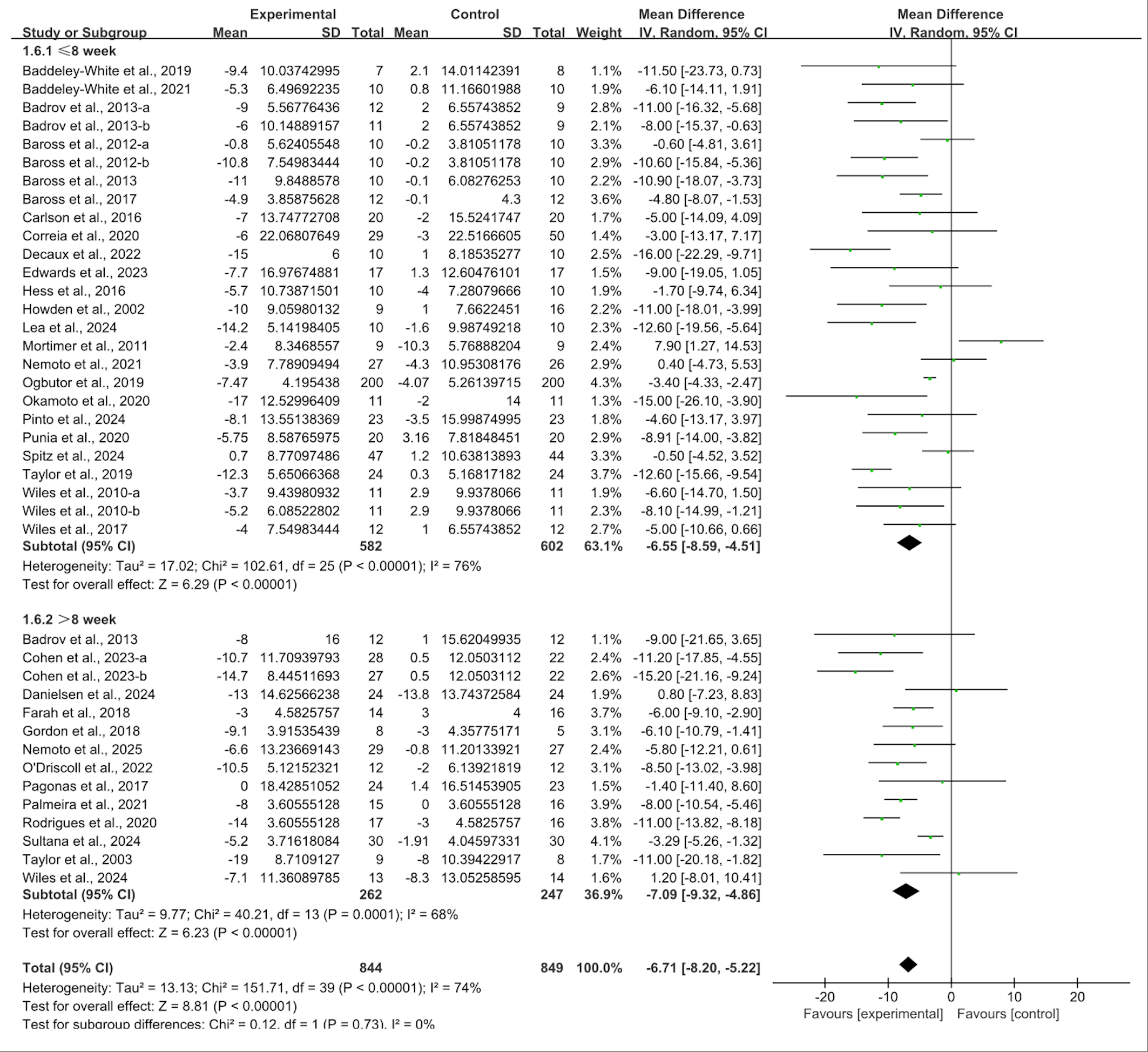

Supplement: Supplementary file 1 [file Supplementary_file_1.zip › Supplementary Materials/Figure 6.tiff]

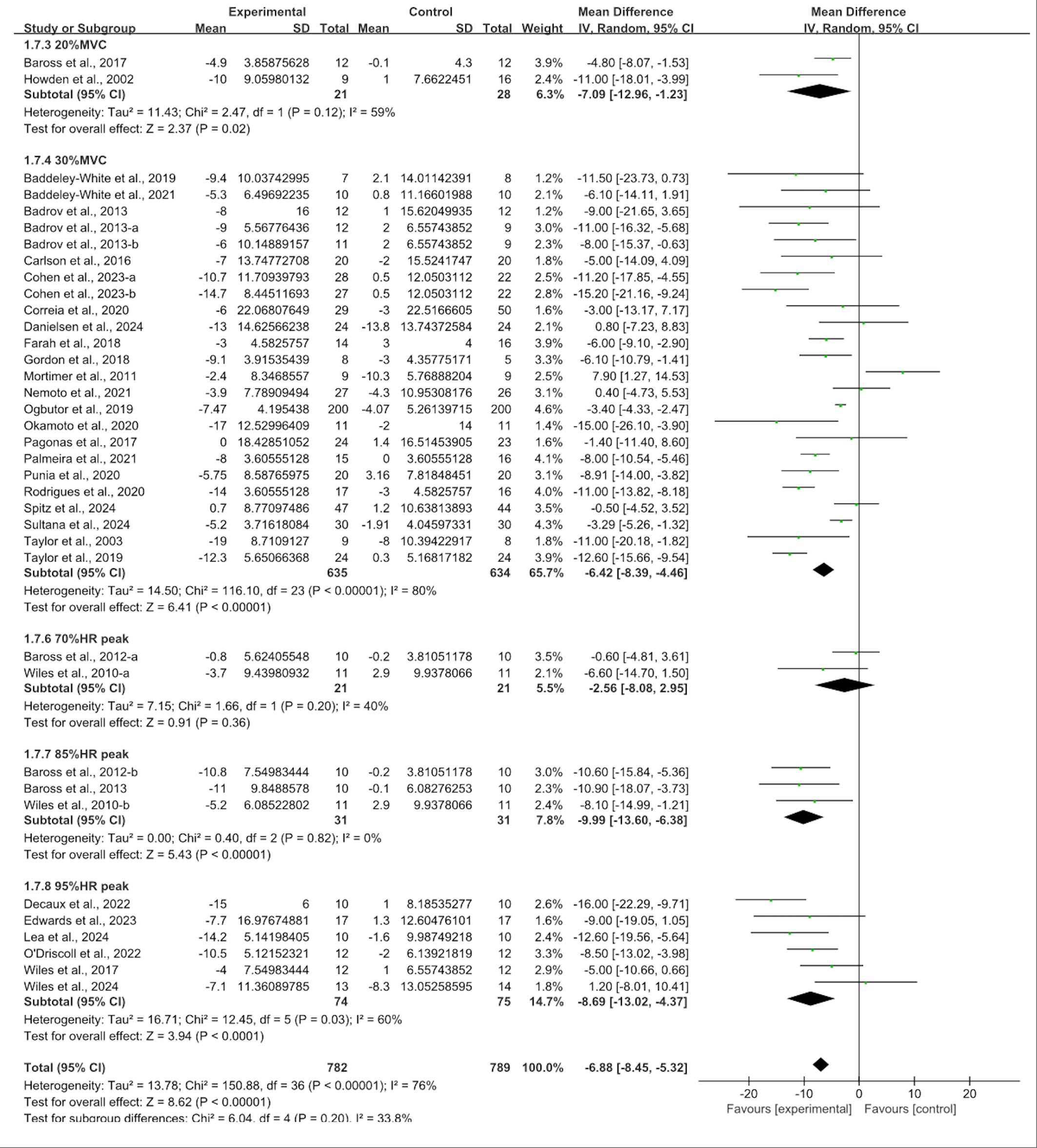

Supplement: Supplementary file 1 [file Supplementary_file_1.zip › Supplementary Materials/Figure 7.tiff]

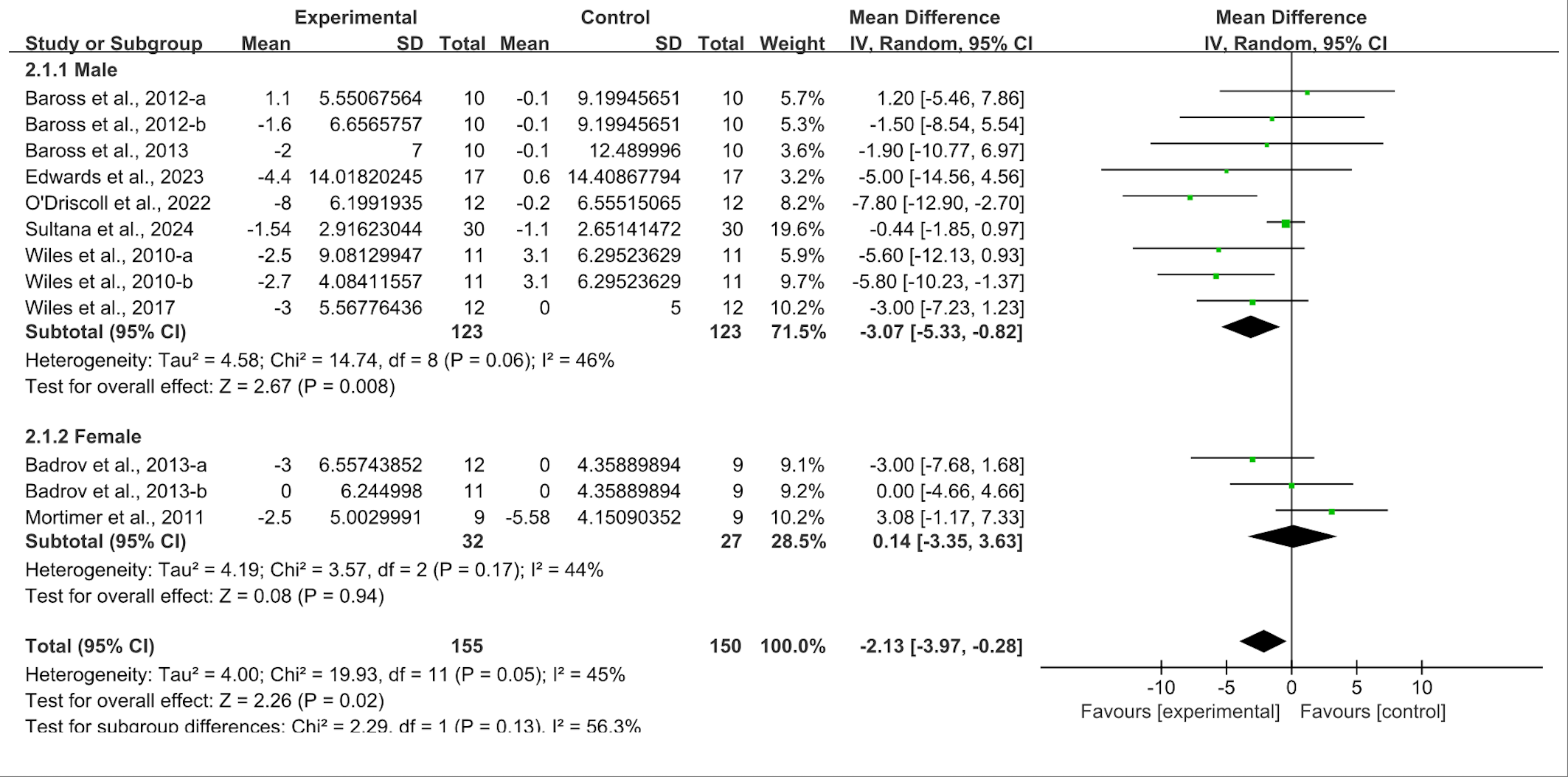

Supplement: Supplementary file 1 [file Supplementary_file_1.zip › Supplementary Materials/Figure 8.tiff]

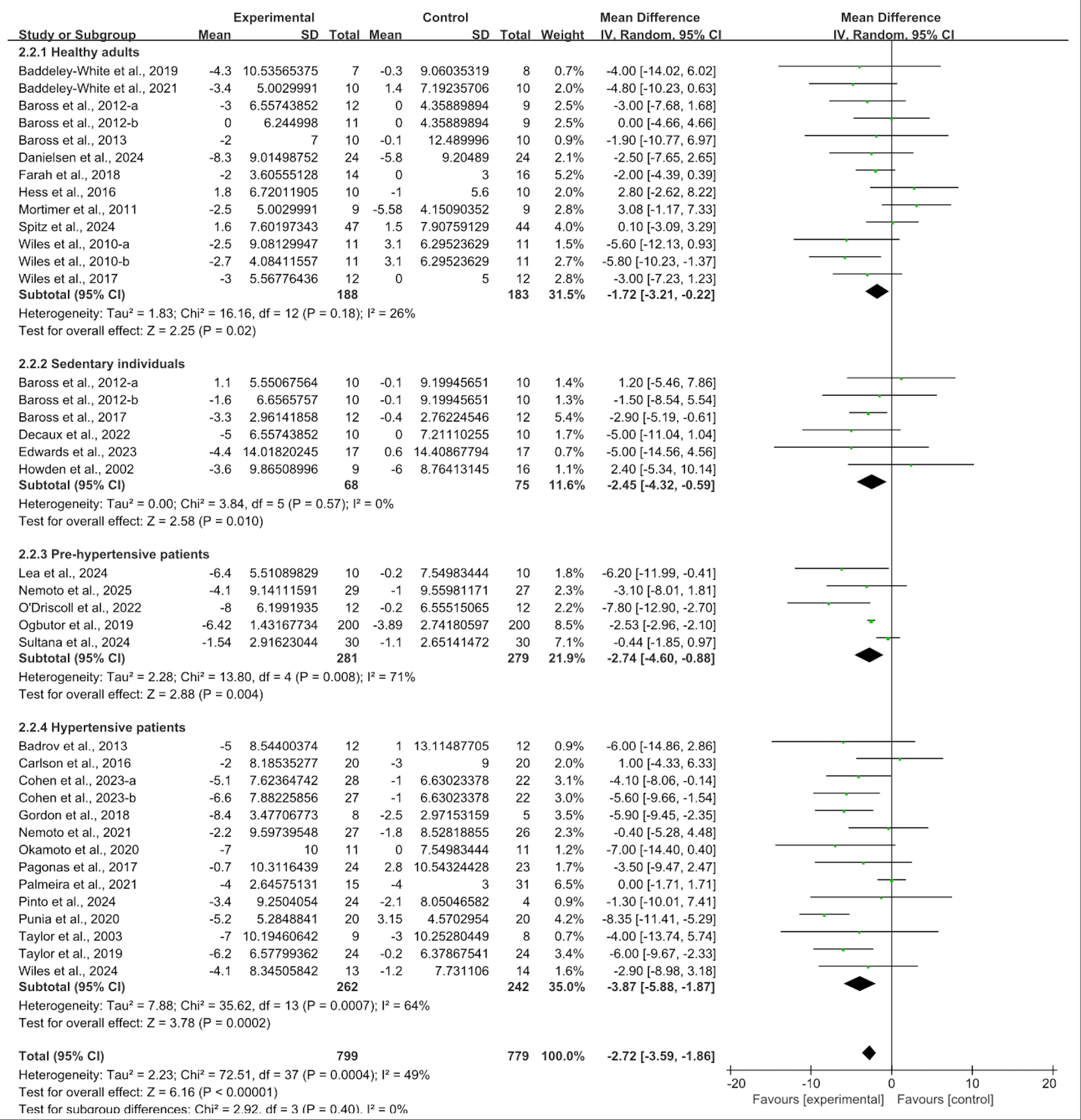

Supplement: Supplementary file 1 [file Supplementary_file_1.zip › Supplementary Materials/Figure 9.tiff]
